# Exploring the chemodiversity of antimicrobial minalemines from *Didemnum granulatum* by neutral loss graph

**DOI:** 10.1038/s41598-025-32070-2

**Published:** 2026-01-07

**Authors:** Vítor F. Freire, Jason R. Evans, Lucero Martínez-Fructuoso, Rohitesh Kumar, Rhone K. Akee, Svetlana Hogan, Christopher C. Thornburg, Brian D. Peyser, Susan Ensel, Dongdong Wang, Tanja Grkovic, Barry R. O’Keefe

**Affiliations:** 1 Natural Products Branch, Developmental Therapeutic Program, Division of Cancer Treatment and Diagnosis, National Cancer Institute, Frederick, MD 21702-1201 USA; 2 Natural Products Support Group, Frederick National Laboratory for Cancer Research, Leidos Biomedical Research, Inc., Frederick, MD 21702-1201 USA; 3 Department of Chemistry and Physics, Hood College, Frederick, MD 21701-8599 USA; 4 Molecular Targets Program, Center for Cancer Research, National Cancer Institute, Frederick, MD 21702-1201 USA

## Abstract

In this study, we report on the identification of the active antimicrobial principles from the tunicate *Didemnum granulatum*. Two new natural products, minalemines G (**1**) and H (**2**), were isolated and their structures were elucidated. Both compounds showed potent antibacterial activity, with compound **1** showing sub-microgram inhibition against *Staphylococcus aureus*. Moreover, we also report a new mass spectrometry-based approach, named neutral loss graph, developed to explore minor metabolites from complex mixtures. This approach was applied here to reveal twelve additional minalemine analogues in the organic extract of *D. granulatum*.

**Supplementary Information:**

The online version contains supplementary material available at 10.1038/s41598-025-32070-2.

## Introduction

Marine organisms are a rich source of bioactive secondary metabolites that have inspired the development of successful drugs based on their natural scaffolds, with a total of 15 compounds approved, and 31 others in various stages of clinical evaluation^1–3^. Three of the approved drugs derived from marine sources were initially identified from tunicates, highlighting the importance of the continue exploration of this subphylum for drug discovery^4^. *Didemnum* is a genus of marine tunicates widely distributed across tropical and subtropical coastal regions of Asia, Oceania, Africa, Europe, and the Americas. Their broad geographical distribution is mirrored by a large chemodiversity of secondary metabolites with over 200 natural products reported, including alkaloids^5^, peptides^6^, polyketides^7^, lipids^8^, steroids^9^ and nucleosides^9,10^. Many of these compounds possess significant biological activity with potential pharmacological applications, such as the antiproliferative lamellarins^11^, the cycle checkpoint inhibitory granulatimides^5^, the HIV protease inhibitory didemnaketals^12^, and the antimalarial and antitrypanosomal lepadins^13^. The enediynes namenamicin and shishijimicins^14^ are potent cytotoxic compounds that closely resemble the microbial natural product calicheamicin, a compound used as an antibody–drug conjugate for treating acute myeloid (Mylotarg®) and lymphocytic leukemias (Besponsa^®^)^10,15^. Overall, although the chemical diversity of tunicates has been extensively studied, there remains significant potential for new discoveries.

Recently, a large antimicrobial high-throughput screen (HTS) was reported by the National Cancer Institute (NCI) and the National Institute of Allergy and Infectious Diseases (NIAID)^16^. The project involved screening 326,000 natural product fractions against four microbial targets:* Staphylococcus aureus*, *Escherichia coli* (including two strains—wild type and *tol*C efflux mutant), and *Candida albicans* and identified over 3,000 fractions with potent antimicrobial activity. From this, 75 active fractions identified during the discovery campaign were selected for further HPLC fractionation and their chemotypes identified using 1 mg of material according to previously published automated methods^17^. HPLC-subfractions from *D. granulatum* showed activity against all four microbial strains (Fig. S1), and using NMR fingerprints, the major chemical components of those fractions were assigned as lipopeptides, although no complete structure was proposed^16^. Herein, we report additional studies on the antimicrobial fractions from *D. granulatum*, describing the isolation and biological evaluation of minalemines G (**1**) and H (**2**), together with an application of neutral loss graph as a tool to explore minor metabolites in the adjacent chemical space.

## Results

### Rapid identification of compounds in bioactive subfractions

**Fig. 1:**
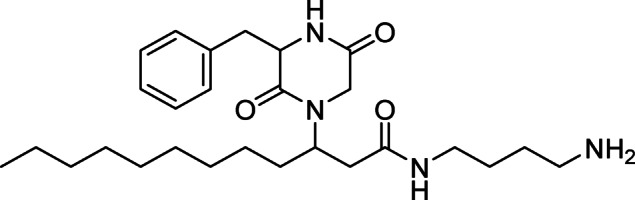
Structure of rodriguesine A. The compound was identified from *D. granulatum* based on an LC–MS dereplication workflow.

Despite the potential utility of compounds derived from tunicates, identifying new chemical entities with pharmacological potential in these organisms can be a laborious process, often complicated by the rediscovery of known metabolites^18^. To address this challenge, LC–MS and taxonomic data have been employed in modern dereplication workflows, utilizing analytical datasets and comprehensive databases for metabolite profiling^19^. As the starting point of this study, we performed a mass spectrometry-based annotation of the constituents in the active subfractions from *D. granulatum*. Initially, the accurate mass of *m/z* features present in the active subfractions of *D. granulatum* were searched against a dataset of 182 compounds from the genus *Didemnum* assembled from the *Dictionary of Natural Products* database^20^. Then, a comparison between the MS^2^ spectra of annotated features and literature reports was used to support dereplication. This approach led us to identify the *m/z* feature at 459 (R_T_ = 4.71 min, subfraction 8) as rodriguesine A (Fig. 1)^21^, supported by comparison with the previously reported fragmentation profile (Fig. S2, Table S1)^22^.

**Fig. 2:**
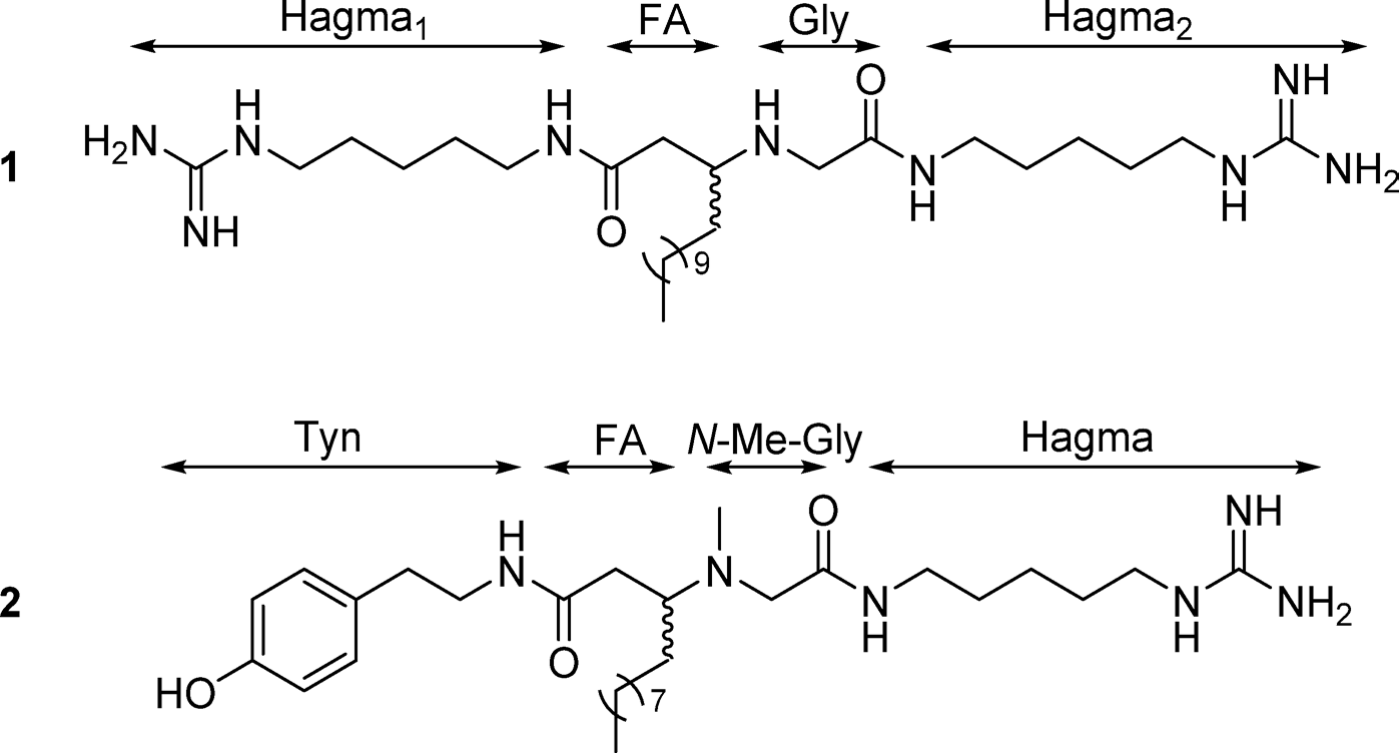
Structures of minalemines G (**1**) and H (**2**). Compound **1** is composed of two homoagmatine (Hagma) subunits, along with fatty acid (FA) and glycine (Gly), while **2** is composed of tyramine (Tyn), FA, *N*-Me-Gly and Hagma.

The strategy successfully used in the annotation of rodriguesine A, which was found exclusively in one of the active subfractions, suggesting that other constituents were responsible for the observed activity. LC–MS traces of the active subfractions revealed that the samples were mixtures of multiple components. Subsequent isolation efforts with additional material from the organic extract of *D. granulatum* led to the purification of two new compounds, minalemines G (**1**) and H (**2**) (Fig. 2).

### Structure elucidation of minalemines G (1) and H (2)

**Fig. 3:**
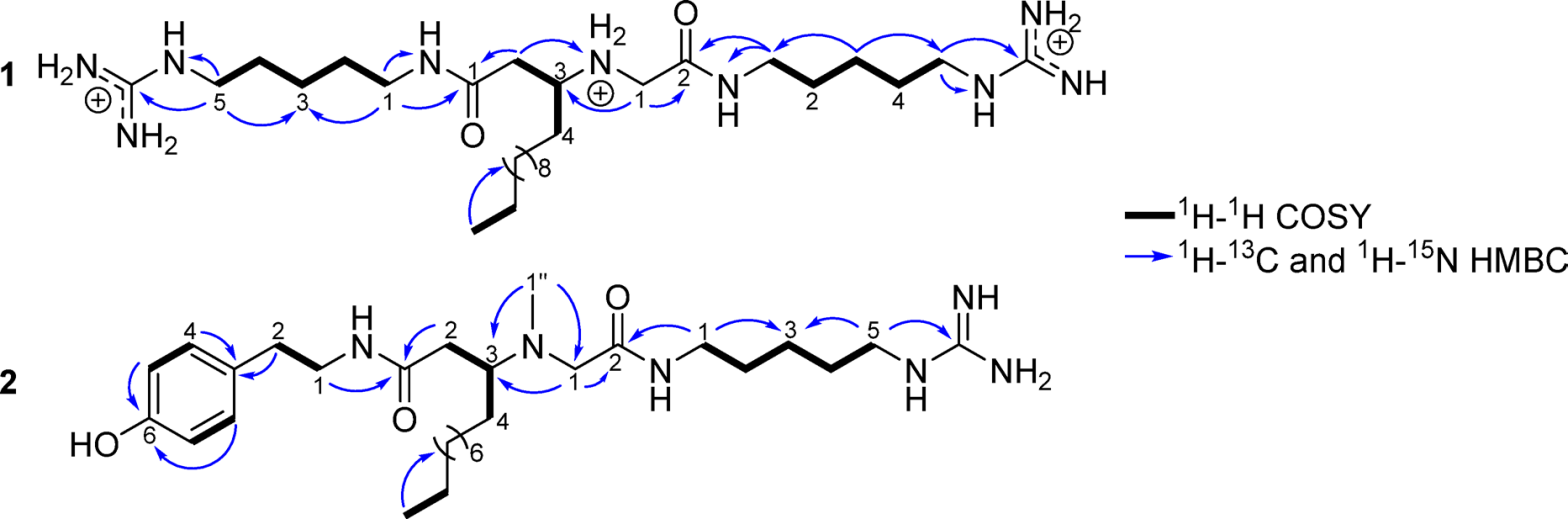
Key ^1^H–^1^H COSY, ^1^H–^13^C and ^1^H–^15^N HMBC correlations of minalemine G (**1**) of the free-base form of minalemine H (**2**).

Minalemine G (**1**) showed a [M + H]^+^ at *m/z* 554.4873 by HRESIMS, corresponding to the molecular formula C_28_H_60_N_9_O_2_^+^. The ^1^H NMR of **1** (Table 1) showed one *sp*^3^ methine at *δ*_H_ 3.52, multiple methylenes ranging between *δ*_H_ 3.88–1.29, and one terminal methyl at *δ*_H_ 0.90. A spin system formed by five methylenes (Hagma_1_-H1–5) was revealed by ^1^H–^1^H COSY correlations (Fig. 3). Analysis of ^1^H–^15^N and ^1^H–^13^C HMBC showed correlations from Hagma_1_-H5 (*δ*_H_ 3.17) to a terminal guanidine (*δ*_N_ 82.9 and *δ*_C_ 158.6), forming the first homoagmatine subunit (Hagma_1_). The methylene at Hagma_1_-H1(*δ*_H_ 3.22) showed correlations to Hagma_1_-N1 (*δ*_N_ 122.7) and to the fatty acid portion (FA) through the carbonyl at FA-C1 (*δ*_C_ 172.4). The second homoagmatine subunit (Hagma_2_) exhibited a similar spin system containing five methylenes with overlapped ^1^H, ^13^C and ^15^N chemical shifts to Hagma_1_, differing at CH_2_-1 (*δ*_H_ 3.27, *δ*_C_ 40.4) and N1 (*δ*_N_ 116.0). Hagma_2_ was connected to the C-terminal of a glycine (Gly) residue by a ^1^H–^13^C HMBC correlation from Hagma_2_-H1 (*δ*_H_ 3.27) to Gly-C2 (*δ*_C_ 166.5). The final planar structure was formed by linking the Gly residue to FA subunit through correlations from Gly-H1 (*δ*_H_ 3.82/3.88) to FA-C3 (*δ*_C_ 57.5), confirmed by the correlation from FA-H2 (*δ*_H_ 2.57/2.70) to Gly-N1 (*δ*_N_ 45.8). With the planar structure established, a competing enantioselective conversion method (CEC)^23^ was applied to determine the absolute configuration of C-1 in **1**, but the kinetics of the reactions using *R*- and *S-*homobenzotetramisole (HBTM) showed no differentiation, suggesting an enantiomeric mixture (Fig. S3). A weak specific rotation ${\left[\alpha \right]}_{\mathrm{D}}^{24}$ of − 2.97 suggested **1** to be a scalemic mixture, which was confirmed by chiral chromatography analysis which showed a 6:4 ratio of the two enantiomers (Fig. S4). Compound **1** was determined to be a new natural product and was named minalemine G.

**Table 1:** NMR data for minalemines G (**1**) and H (**2**) in MeOH-*d*_4_.

**1**^a^				**2**^b^	
Position	*δ*_C_^c^/*δ*_N_^d^, type	*δ*_H_^e^, mult (*J* in Hz)	Position	*δ*_C_^c^/*δ*_N_^d^, type	*δ*_H_^e^, mult (*J* in Hz)
*Hagma*_*1*_			*Tyn*		
1	39.2, CH_2_	3.22, t (7.2)	1	42.3, CH_2_	3.37, td (2.9, 7.1, 7.3)
2	29.9, CH_2_	1.56, m	2	35.6, CH_2_	2.71, t (7.1)
3	24.9, CH_2_	1.40, m	3	131.0, C	
4	29.4, CH_2_	1.61, m	4/8	130.7, CH	7.03, dd (2.2, 8.5)
5	42.3, CH_2_	3.17, t (7.1)	5/7	116.3, CH	6.70, dd (2.2, 8.5)
1-NH	122.7, NH	–	6	157.3, C	–
5-NH	82.9, NH	–	*FA*		
C=N	158.6, C	–	1	175.0, C	–
*FA*			2	37.4, CH_2_	2.24, dd (6.0, 14.4) 2.31, dd (8.8, 14.4)
1	172.4, C	–	3	62.4, CH	2.94, m
2	34.9, CH_2_	2.57, dd (7.2, 16.4) 2.70, dd (4.6, 16.4)	4	30.8, CH_2_	1.27, m 1.55, m
3	57.5, CH	3.52, m	5–11	23.7–33.1, CH_2_	1.29–1.30, m
4	24.3, CH_2_	1.61, m	12	14.4, CH_3_	0.89, t (7.2)
5–13	23.8 – 33.1, CH_2_	1.29–1.40, m	*N-Me-Gly*		
14	14.5, CH_3_	0.90, t (7.0)	1	58.7, CH_2_	3.06, s
*Gly*			2	174.2, C	-
1	46.5, CH_2_	3.82, d (15.5) 3.88, d (15.5)	N-Me	37.4, CH_3_	2.21, s
2	166.5, C	–	*Hagma*		
NH-1	45.8, NH_2_	–	1	39.7, CH_2_	3.23, td (1.8, 7.5)
*Hagma*_*2*_			2	29.4, CH_2_	1.59, m
1	40.4, CH_2_	3.27, t (7.1)	3	24.9, CH_2_	1.39, m
2	29.9, CH_2_	1.56, m	4	30.1, CH_2_	1.56, m
3	25.0, CH_2_	1.40, m	5	42.4, CH_2_	3.16, t (1.8, 7.2)
4	29.4, CH_2_	1.61, m	C=N	158.6, C	–
5	42.3, CH_2_	3.17, t (7.1)			
1-NH	116.0, NH	–			
5-NH	83.0, NH	–			
C=N	158.6, C	–			

^a^TFA salt, ^b^Free base, ^c^151 MHz, ^d^60 MHz, ^e^600 MHz.

**Fig. 4:**
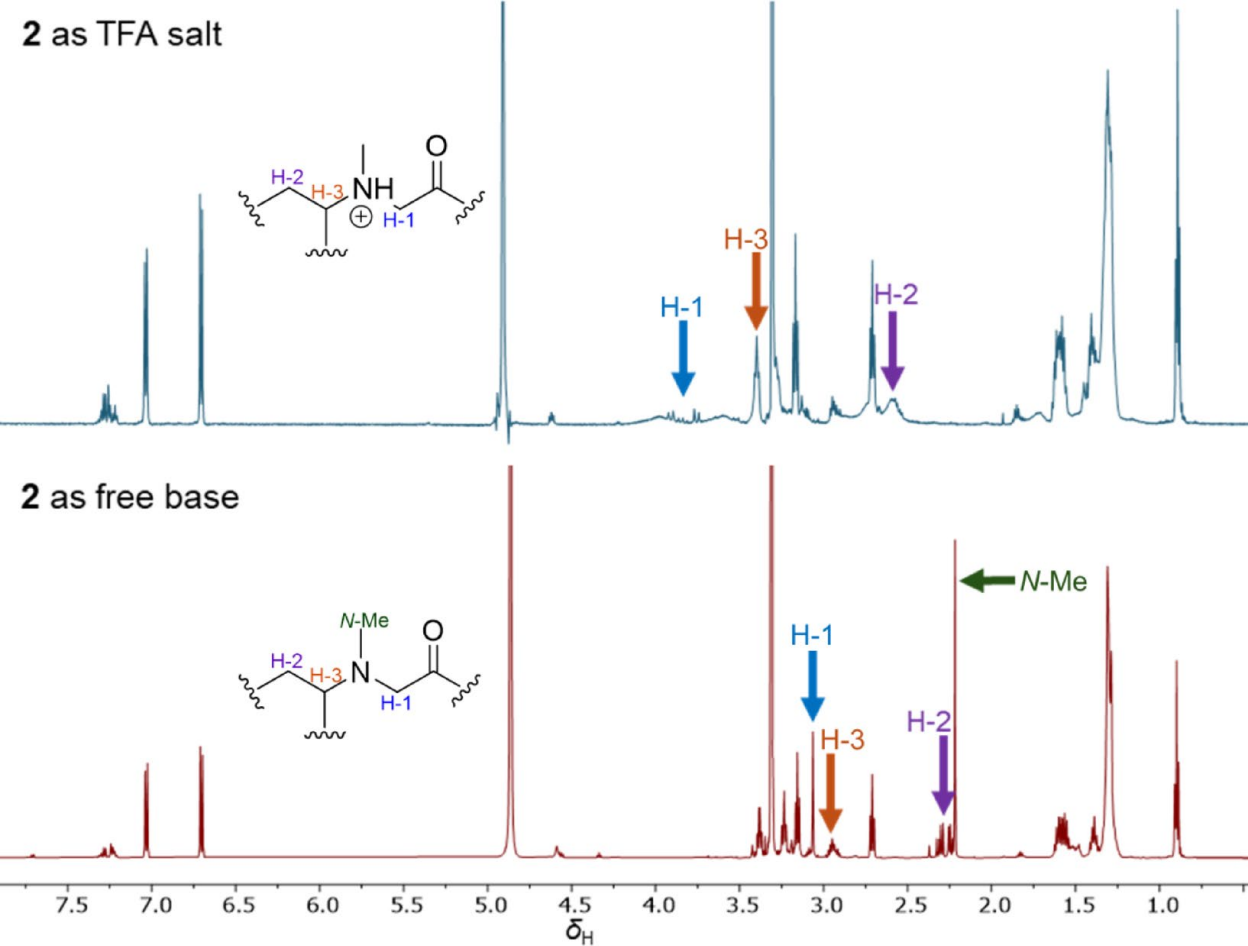
Comparison of signals on ^1^H NMR spectra between TFA salt and free base forms of compound **2** in MeOH-*d*_4_.

Minalemine H (**2**) showed a [M + H]^+^ at *m/z* 533.4172 by HRESIMS, corresponding to the molecular formula C_29_H_53_N_6_O_3_^+^. Analysis of 1D and 2D NMR of the TFA salt form of **2** showed broad signals for the methine and methylene protons of FA and *N*-Me-Gly, resulting in a lack of key correlations for these subunits. Consequently, compound **2** was converted to a free base form by eluting its methanolic solution through an amino SPE cartridge. Figure 4 shows the comparison between the NMR spectra of the TFA salt and free base forms of **2**, highlighting the FA-(*N*-Me-Gly) region. The 1D and 2D NMR data of the free base form of **2** showed clearer signals, multiplicities, and correlations, supporting the structure elucidation outlined below.

NMR data revealed structural resemblance between compounds **1** and **2** by sharing one Hagma and FA subunits. Additionally, the ^1^H NMR spectrum of **2** showed two singlets at *δ*_H_ 3.06 and *δ*_H_ 2.21, with reciprocal ^1^H–^13^C HMBC correlations, forming an *N-*Me-Gly moiety. An 1,4-disubstituted aromatic containing two doublet of doublets at *δ*_H_ 6.70 (Tyn-H5/H7) and *δ*_H_ 7.03 (Tyn-H4/H8) with coupling constants of 2.2 and 8.5 Hz, was connected to two methylenes at Tyn-C2 (*δ*_C_ 35.6) and Tyn-C1 (*δ*_C_ 42.3) through HMBC correlation, establishing a tyramine (Tyn) subunit. The Tyn and FA substructures were connected through correlations between Tyn-H1 (*δ*_H_ 3.37) and FA-H2 (*δ*_H_ 2.24/2.31) to the carbonyl at FA-C1 (*δ*_C_ 175.0). The FA moiety showed HMBC correlations from FA-H3 (*δ*_H_ 2.94) to the methyl and methylene of the *N-*Me-Gly subunit. Additionally, *N-*Me-Gly was attached to Hagma, as shown by correlations from *N-*Me-Gly-H1 (*δ*_H_ 3.06) and Hagma-H1 (*δ*_H_ 3.23) to the carbonyl group at *N-*Me-Gly-C2 (*δ*_C_ 174.2). As observed for **1**, compound **2** also showed a low specific rotation value, ${\left[\alpha \right]}_{\mathrm{D}}^{24}$ of − 4.44. Chiral chromatographic analysis indicated a 7:3 enantiomeric ratio, revealing it as a scalemic mixture. Compound **2** was identified as a new natural product, and named minalemine H.

### Exploring minor metabolites using neutral loss graph method

**Fig. 5:**
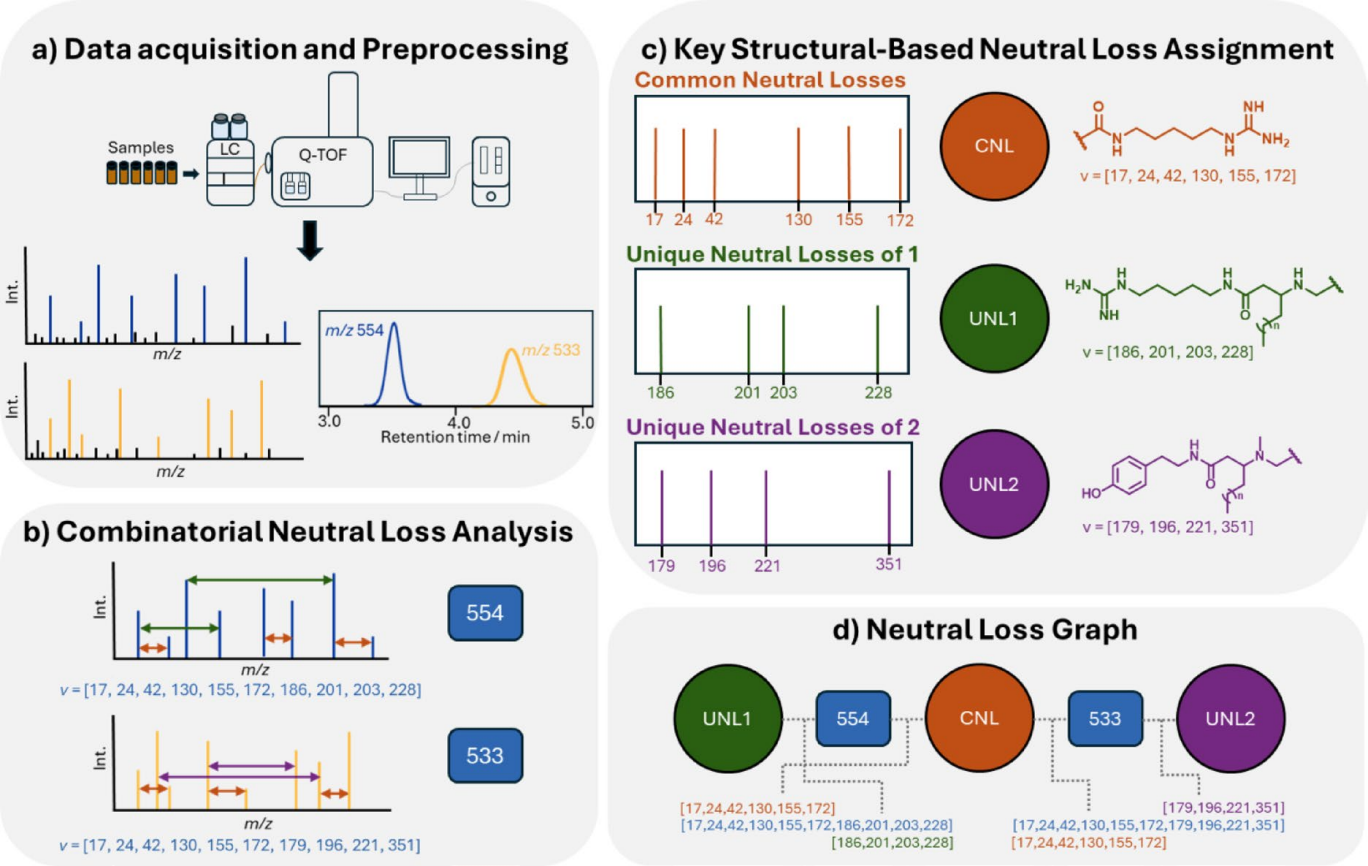
The concept of neutral loss graph (NLG). **a** LC–MS/MS data acquisition and preprocessing combining feature detection and alignment. **b** Combinatorial analysis of experimental neutral losses between all ions in a *m/z* feature. **c** Determination of key structural neutral losses for each core. **d** Calculation of similarity vectors and network construction results in an NLG.

Due to challenges during isolation and limited availability of organic extract material, only compounds **1** and **2** were isolated in sufficient quantities for complete structural characterization by NMR. However, LC–MS analysis of the fractions revealed several additional peaks with similar ionization profiles, suggesting the presence of analogues of **1** and **2**. To explore the chemodiversity of these minor metabolites, we developed the neutral loss graph (NLG), a mass spectrometry-based approach that uses a combinatorial neutral loss analysis to explore structural similarities of closely related compounds. The NLG uses LC-MS^2^ experimental data to generate a list of all existing neutral losses within each *m/z* feature. Then, key neutral losses from the target compounds serve as probes for pair-wise similarity analysis, with the output displayed as a network graph (Fig. 5).

**Fig. 6:**
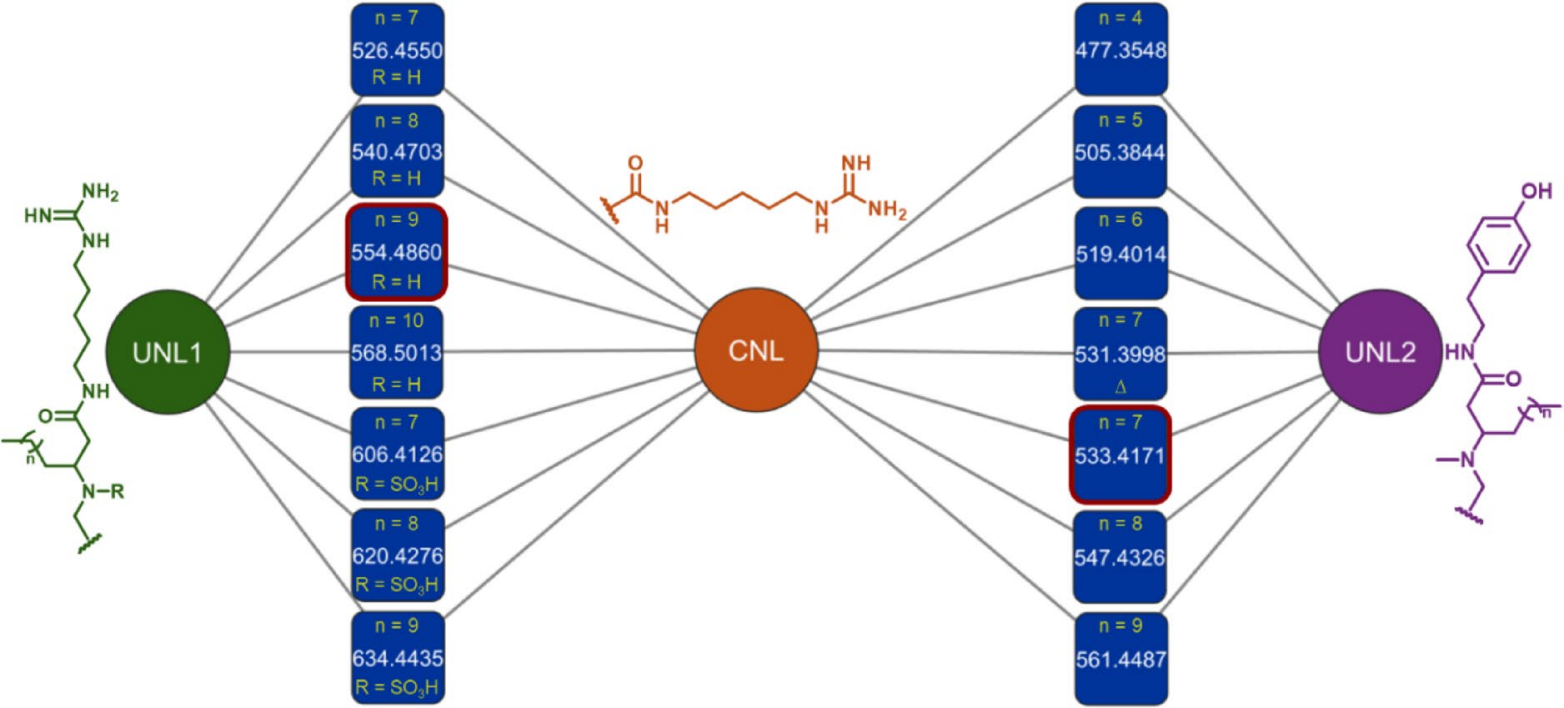
Minalemine’s cluster on the neutral loss graph (NLG). The common neutral loss (CNL) represents the shared subunit of Hagma. The unique neutral loss 1 (UNL1) corresponds to the unique subunits of Hagma-FA-Gly from minalemine G (**1**, node *m/z* 554.4860). While the unique neutral loss 2 (UNL2) is composed by Tyn-FA-*N*-Me-Gly, specific subunits of minalemine H (**2**, node *m/z* 533.4171).

To explore the chemodiversity of *D. granulatum* using NLG method, fractions 1–7, generated from the SPE fractionation, were analyzed by LC-MS^2^ in a data-dependent acquisition mode (DDA). The raw data (.d) was converted to an open-source format (mzML) and preprocessed on MZmine 3.4.27, where the aligned feature list was exported as a mgf file. This file was then used for a combinatorial neutral loss analysis, calculating all-to-all mass differences, from the precursor ion to its fragments, and between fragments within the same *m/z* feature, generating a new mgf file. Parallel to that, three sets of target neutral losses were established: common neutral losses (CNL), unique neutral losses of **1** (UNL1), and **2** (UNL2). The CNL contained six neutral losses (Table S2) related to the Hagma unit found in compounds **1** and **2**, consisting of small mass losses such as NH_3_ and CHN, and medium mass losses, such as C_7_H_16_N_4_O. The UNL1 encompassed four larger neutral losses related to Hagma-FA-Gly, substructures of **1**, while UNL2 was comprised of four large neutral losses related to Tyn-FA-*N*-Me-Gly substructures of **2**. The neutral loss graph was then built by creating a pair-wise similarity matrix, comparing the combinatorial neutral loss of each *m/*z feature and the sets of CNL, UNL1 and UNL2. Figure 6 shows the cluster containing minalemines present in the NLG displayed on Cytoscape, where all features not directly connected to the CNL, and UNL1, or UNL2 were excluded. This cluster showed a total of fourteen *m/z* features with edges connecting them to the CNL and UNL1 or UNL2 (Fig. 6).

Minalemine G (**1**, *m/z* 554) clustered with six other *m/z* features (Fig. 6, Table S3) showing edges to the groups of neutral losses CNL and UNL1, indicating related substructures among them. Analysis of MS^1^ spectra for features *m/z* 526, 540, and 568 showed differences of -28, -14, and + 14 Da, respectively, compared to the monoprotonated molecule [M + H]^+^ of **1**. Characteristic fragments such as [M + H–C_7_H_16_N_4_O]^+^, [M + H—C_8_H_18_N_4_O]^+^, [M + H–C_8_H_19_N_5_O]^+^ and [M + H–C_9_H_20_N_6_O]^+^, suggested these features to be part of a homologous series of **1**, differing only in FA chain length. The MS^2^ data of features *m/z* 606, 620, and 634 showed an additional neutral loss of SO_3_H (79.956 Da), comprising fragment ions at *m/z* 526, 540 and 554 respectively, suggesting their structures to be sulfamic acid-containing compounds, analogous to previously reported minalemines D-F containing sulfamic acid group at Gly-N1^24^.

Minalemine H (**2**, *m/z* 533) shared connection with CNL and UNL2, as well as six other *m/z* features (Fig. 6, Table S3). Analysis of MS^1^ spectrum of features *m/z* 477, 505, 519, 547 and 561 showed differences of -56, -28, -14, + 14, and + 28 Da compared to the monoprotonated molecule [M + H]^+^ of **2**. MS^2^ spectra of these features displayed the same fragmentation pattern as **2**, with characteristic fragment ions such as [M + H–C_10_H_13_NO_2_]^+^, [M + H–C_10_H_16_N_2_O_2_]^+^, [M + H–C_11_H_5_N_3_O_2_]^+^ and [M + H—C_17_H_29_N_5_O_3_]^+^, indicating that these *m/z* features form a homologue series of **2**. Inspection of MS^1^ spectrum of feature *m/z* 531 implied an additional unsaturation compared to **2**. MS^2^ analysis revealed a -2 Da difference for most fragment ions, except for *m/z* 216 [M + H–C_20_H_29_NO_2_]^+^ and *m/z* 199 [M + H–C_20_H_32_N_2_O_2_]^+^, which are fragments without the FA chain, strongly suggesting the position of the unsaturation to be at that subunit.

### Antimicrobial activity

The minimum inhibitory concentration (MIC) of minalemines G (**1**) and H (**2**) were established for various bacterial and fungal strains (Table 2, Fig. S5), which included *E. coli* wild-type (BW25113) and *E. coli tol*C efflux-deficient (JW5503-1), *P. aeruginosa* efflux-mutant (PAM 1626), *S. aureus* wild-type (ATCC 29213), *E. faecalis* wild-type (ATCC29212), vancomycin-resistant *E. faecium* (VRE, ATCC 700221), *C. albicans* (ATCC90028), and *A. fumigatus* (ATCC MYA-3626). Levofloxacin served as positive control for bacterial strains, while amphotericin B was used for the fungal strains. Minalemines G (**1**) and H (**2**) did not show antifungal activity against *C. albicans* or *A. fumigatus*, but both compounds showed activity against *S. aureus*, with MIC values of 0.62 µg/mL for **1** and 2.50 µg/mL for **2**. Furthermore, the Gram-negative *E. coli tol*C efflux-deficient strain was more susceptible to both compounds than the *E. coli* wild type, suggesting these compounds could be substrates of the *tol*C efflux pumps. Minalemine G (**1**) also exhibited a MIC value of 5.00 µg/mL against the efflux mutant strain of *P. aeruginosa*.

**Table 2:** Minimum inhibitory concentration (µg/mL) of minalemines G (**1**) and H (**2**) against selected bacterial and fungal strains.

Microbial strains	1	2	Levofloxacin	Amphoteracin B
*E. coli* (BW25113)	20	> 20	0.03	> 8
*E. coli*, *tol*C efflux deficient (JW5503-1)	1.25	5	0.08	> 8
*P. aeruginosa*, efflux mutant (PAM 1626)	5	> 20	0.015	> 8
*S. aureus* (ATCC 29213)	0.62	2.5	0.12	> 8
*E. faecalis* (ATCC 29212)	20	20	0.5	> 8
*E. faecium*, VRE (ATCC 700,221)	10	20	> 4	> 8
*C. albicans* (ATCC 90,028)	10	> 10	> 2	0.5
*A. fumigatus* (ATCC MYA-3626)	> 10	> 10	> 2	1

## Discussion

Antimicrobial activity guided-isolation of the active principles from the organic extract of the tunicate *D. granulatum* resulted in the purification of two new guanidine alkaloids, named minalemines G (**1**) and H (**2**). Compounds **1** and **2** are new representatives of the minalemine class, previously composed of six compounds, minalemines A-F, isolated from a *D. rodriguesi* collected in New Caledonia^24^. The minalemines G (**1**) and H (**2**) described here differ from those previously reported by the absence of a leucine residue, along with the substitution of the agmatine subunit with homoagmatine in compound **1**, and with tyramine in **2**.

The chemical structure of a compound is directly reflected in fragment ions displayed in its MS^2^ spectrum. Even analogues with subtle structural modifications can exhibit fragments with distinctive *m/z* values^25^. Visualization tools, such as feature-based networks, can explore similarities in MS/MS fragmentation between known and unknown compounds, expanding the annotation and assisting in the dereplication efforts, leading to new discoveries^26,27^. These innovative approaches have facilitated the identification of new compounds at a faster pace, ushering natural products research into a new era^19,26,28^. However, analogues tend to show similar fragmentation pathways, which are disclosed by their neutral losses. Neutral loss has been used for spectral similarity analysis and metabolite annotation^29^, and to enhance molecular similarity analysis in METLIN^25^. Additionally, the use of neutral loss has been shown to outperform the cosine similarity for numerous small molecules^30^. Even though traditional molecular networking approaches combine fragment ions and neutral loss matches using a modified cosine score, it still relies on overlapped fragment ions in order to cluster two compounds together^30^. The minalemines presented no fragment overlap in the MS/MS (Fig. S6), failing to cluster both compounds together. This observation led us to develop the neutral loss graph method, a modified approach that relies on neutral loss analysis to propagate the annotation of target compounds. When applied to *D*. *granulatum* at the fraction level, NLG resulted in the annotation of twelve additional analogues of compounds **1** and **2**. Six analogues of **2** form a homologous series, differing only in the length of the FA moiety. Moreover, *m/z* features annotated as analogues of **1** include a homologue series of minalemine G, and their respective sulfamic acid derivatives. The NLG demonstrates the applicability of neutral loss analysis as an alternative method for exploring a chemical space, leading to the annotation of minor metabolites and providing insights into their structures. Our results demonstrate that integrating LC–MS/MS data into the dereplication workflow is a reliable strategy to reveal minor compounds of interest, opening possibilities for further research to better explore chemical, biological, and pharmacological space.

Minalemines G (**1**) and H (**2**) demonstrated antibacterial activity, with compound **1** showing notable activity against the Gram-positive *S. aureus*. The more potent activity of **1** suggests that the addition of charged sites in its structure enhances its potency. The amphiphilic nature of the minalemines could lead to cell membrane disruption. However, their lower MIC against specific strains makes it reasonable to suggest that these compounds may possess a different mechanism of action^31^. Maccari and collaborators synthesized linear and cyclic di-guanidine compounds that exhibit potent antibacterial activity. Their structure–activity relationship (SAR) findings showed that modifications to the linker, altering the distance between the guanidine groups, along with derivatization by different substituents, significantly change the activity^31^. One of their synthetic compounds comprising two guanidine subunits linked by a chain of two series of eight methylenes with a secondary amine in the center, and a 1-methylenecyclopropyl group on one guanidine moiety (Fig. S7), showed MIC value below 0.125 µg/mL against a strain of Gram-positive bacteria *Streptococcus pyogenes*^31^. These results indicate that SAR studies could enhance the minalemine class as antibacterial leads for further development.

## Methods

### General procedures

Optical rotations were measured on a Rudolph Research Analytical AUTOPOL IV automatic polarimeter with a 0.25 dm path length cell in methanol at 25 °C. UV spectra were recorded as methanol solutions on a Varian Cary 50-Bio UV/Vis spectrophotometer. FTIR spectra were recorded as thin films on a Bruker Alpha II spectrometer. NMR spectra were recorded at 25 °C on either a Bruker Avance III HD spectrometer, equipped with a 5 mm TCI Cryo-Probe Prodigy or a Bruker Avance III spectrometer equipped with a 3 mm TCI cryogenic probe, both operating at a frequency of 600 MHz for the ^1^H nucleus, 151 MHz for the ^13^C nucleus, and 60 MHz for the ^15^N nucleus. For the 3 mm TCI cryogenic probe, all 2D NMR experiments were acquired with non-uniform sampling (NUS) set to 25% using the standard Bruker pulse sequences. For the 5 mm TCI cryogenic probe, all 2D NMR experiments were acquired with non-uniform sampling (NUS) set to 40% for ^1^H‒^1^H detected experiments or 35% for ^1^H‒^13^C detected experiments using the standard Bruker pulse sequences. Spectra were calibrated to residual solvent signals at *δ*_H_ 3.31 and *δ*_C_ 49.0 for MeOH-*d*_4_ or *δ*_H_ 2.50 and *δ*_C_ 39.5 for DMSO-*d*_6_. The *δ*_N_ values were not calibrated to an external standard but were referenced to neat NH_3_ (*δ*_N_ 0.00) using the standard Bruker parameters. NMR FID processing and data interpretation was done using MestReNova software, version 15.0. Semi-preparative scale HPLC purification was performed with a Gilson HPLC purification system equipped with a GX-281 liquid handler, a 322-binary pump, and a 172-photodiode array detector. All solvents used for chromatography and UV were HPLC grade, and H_2_O was Millipore Milli-Q PF filtered.

High-resolution mass spectra were recorded on an Agilent 1260 Infinity II UHPLC system coupled to an Agilent 6545 QToF equipped with a dual AJS ESI source. A Kinetex C_18_ column (50.0 × 2.1 mm, 1.7 *μ*m, 100 Å Phenomenex) was used. The mobile phase consisted of H_2_O + 0.1% formic acid (A) and MeCN + 0.1% formic acid (B) at a flow rate of 0.7 mL/min. The gradient used was maintained at 95:5 (A:B) for 0.5 min, from 95:5 to 0:100 (A:B) for 8.0 min, maintained at 0:100 (A:B) for 0.5 min, from 0:100 to 95:5 (A:B) for 0.5 min and then equilibrated in a post-run at 95:5 (A:B) during 1.0 min. The positive mode ESI conditions were 3 kV of capillary voltage, 1 kV of nozzle voltage, gas temperature at 300 ˚C and gas flow of 10 L/min. The data dependent acquisition (DDA) mode was used, under the following parameters: positive ionization; acquisition time from 0.5 to 9 min; *m/z* range 100–3200; scan time of 0.5 s; isolation width ~ 4 amu; precursor selection of 10^5^ absolute intensity; 3 MS/MS per MS survey; mass exclusion real time for 0.15 min; and ramp of collision energy for MS/MS 20–30 eV for the lowest mass (*m/z* 200), 40 eV for *m/z* 800, 50–70 eV for *m/z* 1500, and 50–70 eV for the highest mass (*m/z* 3000).

### Collection, extraction, and isolation

The tunicate *D. granulatum* was collected in a depth range of 8–13 m in Northern Australia in September 2002 by the Museum & Art Gallery of the Northern Territory under contract to the Natural Products Branch, Developmental Therapeutics Program, Division of Cancer Treatment and Diagnosis, NCI. The specimen was taxonomically identified by Dr. Patricia Kott Mather, and a voucher (0M9H2221) was deposited at the Smithsonian Institution. The ascidian (wet weight 1110 g) was extracted in water, followed by a MeOH/DCM overnight soak according to the Natural Products Branch’s standard marine extraction procedure^32^, to provide 6.17 g of the organic extract (NSC #C23983). A portion of the organic extract C23983 (3.5 g) was prefractionated on a C_8_ SPE column (20 g), generating seven fractions^33^: 95:5 H_2_O:MeOH (C23983_1), 80:20 H_2_O:MeOH (C23983_2), 60:40 H_2_O:MeOH (C23983_3), 40:60 H_2_O:MeOH (C23983_4), 20:80 H_2_O:MeOH (C23983_5), 0:100 H_2_O:MeOH (C23983_6), 50:50 MeCN:MeOH (C23983_7).

Fractions C23983_3–7 were combined (1.7 g, named C23983_3) and subjected to five HPLC separations using mobile phase of H_2_O + 0.5% TFA (solvent A) and MeCN + 0.5% TFA (solvent B). (*I*) HPLC separation using a Kinetex C_8_ column (150 × 21.2 mm, 5 *μ*m, 100 Å, Phenomenex) on a gradient of 95:5 to 0:100 (A:B) for 23 min at 9.0 mL/min, and fraction collection was performed in 0.33 min increments. HPLC fractions 30–49 (159 mg, named C23983_3_30) were combined and subjected to (*II*) separation using Luna C_4_(2) column (250 × 10 mm, 5 *μ*m, 100 Å, Phenomenex) on a gradient of 68:32 to 50:50 (A:B) for 34 min at 3.8 mL/min, and fraction collection was performed in 0.50 min increments. HPLC fractions 25 – 50 (53 mg, named C23983_3_30_25) were combined and subjected to (*III*) separation using Luna C_4_(2) column (250 × 10 mm, 5 *μ*m, 100 Å, Phenomenex) on a gradient of 67:33 to 55:45 (A:B) for 32 min at 3.8 mL/min, and fraction collection was performed in 0.33 min increments. HPLC fractions 45–49 (5.5 mg, named C23983_3_30_25_45) were combined and subjected to (*IV*) separation using Luna C_4_(2) column (250 × 10 mm, 5 *μ*m, 100 Å, Phenomenex) on a gradient of 65:35 to 62:38 (A:B) for 31 min at 3.8 mL/min, and fraction collection was performed in 0.33 min increments yielding minalemine G (3.4 mg, 0.10% of organic extract yield; **1**). HPLC fractions from separation (*III*) 55–59 (4.6 mg, named C23983_3_30_25_55) were combined and subjected to (*V*) separation using Luna C_4_(2) column (250 × 10 mm, 5 *μ*m, 100 Å, Phenomenex) on a gradient of 65:35 to 60:40 (A:B) for 29 min at 3.8 mL/min, and fraction collection was performed in 0.33 min increments yielding minalemine H (3.9 mg, 0.11% of organic extract yield; **2**).

*Minalemine G (****1****)*: colorless oil, ${\left[\alpha \right]}_{\mathrm{D}}^{24}$ − 2.97 (*c* 0.31, MeOH); UV (MeOH) *λ*_max_ (log *ε*) 220 (5.67) nm; IR (film) *ν*_max_ 3268, 3182, 2926, 2857, 1648, 1549, 1524, 1454, 1368, 1235, 1191, 828, 709 cm^-1^ (Fig. S8); ^1^H and ^13^C data in MeOH-*d*_4_ and DMSO-*d*_6_ are in Tables 1 and S4 and in Figs. S9–S28; HRESIMS *m/z* 554.4873 [M + H]^+^ (calcd. for C_28_H_60_N_9_O_2_^+^ *m/z* 554.4865, Δ = 1.44 ppm, Figs. S29–S30).

*Minalemine H (****2****)*: colorless oil, ${\left[\alpha \right]}_{\mathrm{D}}^{24}$ − 4.44 (*c* 0.45, MeOH); UV (MeOH) *λ*_max_ (log *ε*) 220 (5.87), 278 (5.12) nm; IR (film) *ν*_max_ 3339, 3292, 3193, 3112, 2928, 2857, 1669, 1576, 1435, 1202, 1184, 1136, 839, 802, 723 cm^-1^ (Fig. S31); ^1^H and ^13^C data in MeOH-*d*_4_ and DMSO-*d*_6_ are Tables 1 and S4 and in Figs. S32–S47; HRESIMS *m/z* 533.4172 [M + H]^+^ (calcd. for C_29_H_53_N_6_O_3_^+^ *m/z* 533.4174, Δ = − 0.37 ppm, Figs. S48–S49).

### Compound identification of active subfraction using LC–MS/MS data

The UHPLC-MS/MS data from active subfractions C23983_4_1 to 9 was acquired using the method described above in the general procedures section. The raw data files (.d) were converted to mzML files by MSConvert 3.0.22 (ProteoWizard), with binary encoding precision 64-bit, write index and applied filter of peak picking MS levels 1–2. The mzML files were uploaded to MZmine 3.4.27^34^. Mass detection for MS^1^ and MS^2^ was performed using the mass detector centroid and noise level of 1.0E2. Chromatograms were reconstructed using the function ADAP Chromatogram Builder with retention time between 1.0 and 8.5 min, MS level 1, min group size in # of scans 2, group highest intensity 1.0E5, minimum height 1.0E5 and *m/z* tolerance of 0.005 *m/z* or 20.0 ppm. Deconvolution was performed using the function “group MS^2^ scans with features” with MS^1^ to MS^2^ precursor tolerance of 0.005 *m/z* or 20.0 ppm and retention time filter of 0.50 min. The isotopic peak grouper function was applied with *m/z* tolerance of 0.005 *m/z* or 20.0 ppm, retention time tolerance of 0.50 min, maximum charge of 3, and most intense peak as the representative isotope. Data were aligned using the function join aligner, with *m/z* tolerance of 0.005 m/z or 20.0 ppm, weight for *m/z* of 75, R_T_ tolerance of 0.5 min and weight for R_T_ of 25. The feature list generated after the described steps was exported using the function Export/Submit to GNPS, generating one mgf and one csv file.

The generated csv file was used to search against an *in-silico* library comprising 182 compounds from the genus *Didemnum* assembled from the *Dictionary of Natural Products* database. First, the accurate mass of *m/z* features present in the active subfractions of *D. granulatum* was searched against an *in-silico* library comprising protonated molecules and sodium adducts with exact mass (± 20 ppm). A comparison between the MS^2^ spectrum of annotated features and literature reports was then used to support the annotation.

### Competing enantioselective conversion (CEC)

The CEC reaction followed by LC–MS analysis was based on a previously described procedure^23,35^. Compound **1** (0.20 mg, 0.36 *μ*mol) was transferred to two different vials, and dimethylformamide (90 *μ*L) was added as an organic solvent. The catalysts *S*- and *R*-HBTM (5.0 *μ*L, 4.3 *μ*mol) were added to their respective vials, along with *N*,*N*-diisopropylethylamine (0.7 *μ*L, 4.3 *μ*mol). Propionic anhydride (0.5 *μ*L, 4.3 *μ*mol) was added to initiate the reaction. Aliquots of 2 *μ*L were taken every 5 min and quenched with 100 *μ*L of MeOH prior to LC–MS analysis, for a total reaction time of 60 min.

Aliquots (5 *μ*L) of the samples collected at different time intervals were injected on an Agilent 1260 Infinity II HPLC system coupled to an Agilent 6230 ToF equipped with a dual AJS ESI source. A Kinetex C_18_ column (50.0 × 2.1 mm, 5 *μ*m, Phenomenex) was used for the analysis. The mobile phase consisted of H_2_O + 0.1% formic acid (A) and MeCN + 0.1% formic acid (B) at a flow rate of 1 mL/min. The gradient used was maintained at 95:5 (A:B) for 1.0 min, from 95:5 to 0:100 (A:B) for 8.0 min, maintained at 0:100 (A:B) for 1.0 min, from 0:100 to 95:5 (A:B) for 0.5 min and then equilibrated in a post-run at 95:5 (A:B) during 1.5 min. The positive mode ESI conditions were 3.5 kV of capillary voltage, 1.5 kV of nozzle voltage, gas temperature at 325 °C and gas flow of 10 L/min. MS spectra were acquired using positive mode, mass range of *m/z* 100‒3000, and scan rate of 1 spectra/s. The reaction rates were determined by measuring the peak areas of the fully acylated derivative of compound **1**, using the extracted ion chromatogram (EIC) for the sodiated molecule [M + Na]^+^ at *m/z* 722.5651 (± 20 ppm) on MassHunter Qualitative Analysis 10.0 software.

### Chiral chromatography

The chiral chromatography analysis were performed on a Shimadzu system equipped with a CBM-40 controller, an SPD-M40 PDA detector, and two LC-20AR pumps. A Lux amylose-3 column (250.0 × 4.6 mm, 5 *μ*m, Phenomenex) was used for the analysis. The mobile phase consisted of H_2_O + 0.1% TFA (A) and MeCN + 0.1% TFA (B) using an isocratic mode at 80% of B at a flow rate of 1 mL/min. The injection volume was 5 µL.

### LC–MS/MS analysis and data processing for creating the neutral loss graph

UHPLC-MS/MS data on the scale-up fractions after C_8_ SPE (C23983_1–7) was acquired, converted to mzML, and processed using MZmine using the same parameters as described in the compound identification section. The mgf file was used for a combinatorial neutral loss analysis within each MS^2^ spectrum, where the mass difference was calculated for the precursor ion and its fragments, and between fragments within the same feature. The combinatorial analysis generated a mgf file containing neutral losses for each *m/z* feature.

Three sets of target neutral losses were established, namely common neutral losses (CNL), and unique neutral losses of **1** (UNL1) and **2** (UNL2). The CNL contained six neutral losses from the Hagma unit in compounds **1** and **2** (Table S2). The UNL1 is constituted by four larger neutral losses from Hagma-FA-Gly, substructures of **1** (Table S2), while UNL2 is composed of four neutral losses from Tyn-FA-*N*-Me-Gly, substructures of **2** (Table S2).

### Neutral loss graph package

A simplified version of the molecular networking algorithm^36^ was used to generate a neutral loss graph. The similarity of two features was based on the intersection of their respective neutral loss vectors. Neutral losses for a given feature were derived from the all-to-all differences of the MS2 peak. Given two features with neutral loss vectors *v*_1_ and *v*_2_, the similarity between features was calculated as:

$$0 \le {\mathrm{max}}\left( {\frac{{\sum_{{x \in \left( {v_{1} \cap v_{2} } \right)}}^{x} }}{{\sum_{{x \in v_{1} }}^{x} }}, \frac{{\left| {v_{1} \cap v_{2} } \right|}}{{\left| {v_{1} } \right|}}} \right) \le 1$$

A pair wise matrix of all similarities (1 representing a complete or proper subset) was used as input for generating an undirected adjacency graph. The resultant graph object was exported as a graphml file to be imported and visualized using Cytoscape 3.9.1^37^ (Fig. S50). The algorithm implementation was written using the R statistical computing software (version 4.3.0)^38^. The MSnbase package^39,40^ was used to process mgf files and the igraph package^41^ to build the network and generate the graphml file. Code is available on GitHub at https://github.com/NCI-DCTD/neutralLossGraph.

### Antimicrobial assay

Microbes, growth, and testing conditions were identical to those reported previously for the primary screen^16^.

## Supplementary Information

Supplementary Information.

## Data Availability

The data that support the findings of the present study are available in the SI of this article. In addition, the raw HRMS and NMR data have been deposited in the Harvard Dataverse (dataverse.harvard.edu) and can be found at [10.7910/DVN/W3VBPS]).
