# Supplementary material for: Exploring the chemodiversity of antimicrobial minalemines from Didemnum granulatum by neutral loss graph
Source: Sci Rep. 2026 Jan 7;16:2311. doi: 10.1038/s41598-025-32070-2 (PMC12816755; doi:10.1038/s41598-025-32070-2)
Supplement: Supplementary file 1 — Supplementary Information. [file 41598_2025_32070_MOESM1_ESM.docx]

**Supplementary Information**

**for**

**Exploring the Chemodiversity of Antimicrobial Minalemines from *Didemnum granulatum* by Neutral Loss Graph**

Vítor F. Freire,*^a^ Jason R. Evans,^a^ Lucero Martínez-Fructuoso,^a^ Rohitesh Kumar,^b^ Rhone K. Akee,^b^ Svetlana Hogan,^b^ Christopher C. Thornburg,^b^ Brian D. Peyser,^a^ Susan Ensel,^b,c^ Dongdong Wang,^d^ Tanja Grkovic,^a,d^ Barry R. O’Keefe*^a,d^

^a^Natural Products Branch, Developmental Therapeutic Program, Division of Cancer Treatment and Diagnosis, National Cancer Institute, Frederick, Maryland 21702-1201, United States

^b^Natural Products Support Group, Leidos Biomedical Research, Inc., Frederick National Laboratory for Cancer Research, Frederick, Maryland 21702-1201, United States

^c^Department of Chemistry and Physics, Hood College, Frederick, Maryland 21701-8599, United States

^d^Molecular Targets Program, Center for Cancer Research, National Cancer Institute, Frederick, Maryland

**LIST OF FIGURES**

[**Fig. S1**. Screening of subfractions generated for the antimicrobial campaign between NIAID and NCI.^1^ 4](#_Toc215653263)

[**Fig. S2.** MS^2^ spectrum extracted from the feature: *m/z* 459.3328, R_T_ 4.65 min. 5](#_Toc215653264)

[**Fig. S3.** Competing enantioselective conversion (CEC) reaction with *R*- and *S*-homobenzotetramisole (HBTM) coupled to LC-MS. Reaction rates of **1** treated with propionic anhydride using *R*- and *S*-HBTM catalysts. 7](#_Toc215653265)

[**Fig. S4.** Chromatogram of chiral analysis for minalemines G (**1**) and H (**2**). 8](#_Toc215653266)

[**Fig. S5.** Dose-response graphs of antimicrobial activity for minalemines G (a) and H (b). 11](#_Toc215653267)

[**Fig. S6.** Mirror plot of MS/MS data of minalemines G (**1**, top) and H (**2**, bottom). 12](#_Toc215653268)

[**Fig. S7.** Synthetic compound reported by Maccari *et al*.^2^ 12](#_Toc215653269)

[**Fig. S8.** FTIR spectrum of minalemide G (**1**). 14](#_Toc215653270)

[**Fig. S9.** 600 MHz ^1^H NMR spectrum of minalemide G (**1**) in MeOH-*d*_4_. 15](#_Toc215653271)

[**Fig. S10**. Expansion of 600 MHz ^1^H NMR spectrum of minalemide G (**1**) in MeOH-*d*_4_. 16](#_Toc215653272)

[**Fig. S11**. Expansion of 600 MHz ^1^H NMR spectrum of minalemide G (**1**) in MeOH-*d*_4_. 17](#_Toc215653273)

[**Fig. S12**. Expansion of 600 MHz ^1^H NMR spectrum of minalemide G (**1**) in MeOH-*d*_4_. 18](#_Toc215653274)

[**Fig. S13.** Expansion of 600 MHz ^1^H NMR spectrum of minalemide G (**1**) in MeOH-*d*_4_. 19](#_Toc215653275)

[**Fig. S14**. 151 MHz ^13^C NMR spectrum of minalemide G (**1**) in MeOH-*d*_4_ 20](#_Toc215653276)

[**Fig. S15**. ^1^H–^13^C HSQC NMR spectrum of minalemide G (**1**) in MeOH-*d*_4_ 21](#_Toc215653277)

[**Fig. S16**. ^1^H–^1^H COSY NMR spectrum of minalemide G (**1**) in MeOH-*d*_4_. 22](#_Toc215653278)

[**Fig. S17**. ^1^H–^13^C HMBC NMR spectrum of minalemide G (**1**) in MeOH-*d*_4_ 23](#_Toc215653279)

[**Fig. S18.** ^1^H–^15^N HMBC NMR spectrum of minalemide G (**1**) in MeOH-*d*_4_ 24](#_Toc215653280)

[**Fig. S19.** 600 MHz ^1^H NMR spectrum of minalemide G (**1**) in DMSO-*d*_6_ 25](#_Toc215653281)

[**Fig. S20.** Expansion of 600 MHz ^1^H NMR spectrum of minalemide G (**1**) in DMSO-*d*_6_. 26](#_Toc215653282)

[**Fig. S21.** Expansion of 600 MHz ^1^H NMR spectrum of minalemide G (**1**) in DMSO-*d*_6_. 27](#_Toc215653283)

[**Fig. S22.** Expansion of 600 MHz 1H NMR spectrum of minalemide G (**1**) in DMSO-*d*_6_. 28](#_Toc215653284)

[**Fig. S23.** 151 MHz ^13^C NMR spectrum of minalemide G (**1**) in DMSO-*d*_6_ 29](#_Toc215653285)

[**Fig. S24.** ^1^H–^13^C HSQC NMR spectrum of minalemide G (**1**) in DMSO-*d*_6_. 30](#_Toc215653286)

[**Fig. S25.** ^1^H–^1^H COSY NMR spectrum of minalemide G (**1**) in DMSO-*d*_6_ 31](#_Toc215653287)

[**Fig. S26.** ^1^H–^13^C HMBC NMR spectrum of minalemide G (**1**) in DMSO-*d*_6_ 32](#_Toc215653288)

[**Fig. S27.** ^1^H–^15^N HSQC NMR spectrum of minalemide G (**1**) in DMSO-*d*_6_ 33](#_Toc215653289)

[**Fig. S28.** ^1^H–^15^N HMBC NMR spectrum of minalemide G (**1**) in DMSO-*d*_6_ 34](#_Toc215653290)

[**Fig. S29**. HRESIMS spectrum of minalemide G (**1**). 35](#_Toc215653291)

[**Fig. S30**. Expansion of HRESIMS spectrum of minalemide G (**1**). 35](#_Toc215653292)

[**Fig. S31.** FTIR spectrum of minalemine H (**2**). 36](#_Toc215653293)

[**Fig. S32.** 600 MHz ^1^H NMR spectrum of minalemide H (**2**) in MeOH-*d*_4_ 37](#_Toc215653294)

[**Fig. S33.** Expansion of 600 MHz ^1^H NMR spectrum of minalemide H (**2**) in MeOH-*d*_4_. 38](#_Toc215653295)

[**Fig. S34.** Expansion of 600 MHz ^1^H NMR spectrum of minalemide H (**2**) in MeOH-*d*_4_. 39](#_Toc215653296)

[**Fig. S35.** Expansion of 600 MHz ^1^H NMR spectrum of minalemide H (**2**) in MeOH-*d*_4_. 40](#_Toc215653297)

[**Fig. S36.** 151 MHz ^13^C NMR spectrum of minalemide H (**2**) in MeOH-*d*_4_ 41](#_Toc215653298)

[**Fig. S37.** ^1^H–^13^C HSQC NMR spectrum of minalemide H (**2**) in MeOH-*d*_4_ 42](#_Toc215653299)

[**Fig. S38.** ^1^H–^1^H COSY NMR spectrum of minalemide H (**2**) in MeOH-*d*_4_ 43](#_Toc215653300)

[**Fig. S39.** ^1^H–^13^C HMBC NMR spectrum of minalemide H (**2**) in MeOH-*d*_4_ 44](#_Toc215653301)

[**Fig. S40.** 600 MHz ^1^H NMR spectrum of minalemide H (**2**) in DMSO-*d*_6_ 45](#_Toc215653302)

[**Fig. S41.** Expansion of 600 MHz ^1^H NMR spectrum of minalemide H (**2**) in DMSO-*d*_6_ 46](#_Toc215653303)

[**Fig. S42.** Expansion of 600 MHz ^1^H NMR spectrum of minalemide H (**2**) in DMSO-*d*_6_ 47](#_Toc215653304)

[**Fig. S43.** Expansion of 600 MHz ^1^H NMR spectrum of minalemide H (**2**) in DMSO-*d*_6_. 48](#_Toc215653305)

[**Fig. S44.** 151 MHz ^13^C NMR spectrum of minalemide H (**2**) in DMSO-*d*_6_ 49](#_Toc215653306)

[**Fig. S45.** ^1^H–^13^C coupled HSQC NMR spectrum of minalemide H (**2**) in DMSO-*d*_6_ 50](#_Toc215653307)

[**Fig. S46.** ^1^H–^1^H COSY NMR spectrum of minalemide H (**2**) in DMSO-*d*_6_ 51](#_Toc215653308)

[**Fig. S47.** ^1^H–^13^C HMBC NMR spectrum of minalemide H (**2**) in DMSO-*d*_6_ 52](#_Toc215653309)

[**Fig. S48.** HRESIMS spectrum of minalemide H (**2**). 53](#_Toc215653310)

[**Fig. S49.** Expansion of HRESIMS spectrum of minalemide H (**2**). 53](#_Toc215653311)

[**Fig. S50.** Unfiltered neutral loss graph (NLG) of *Didemnum granulatum* fractions*.* 53](#_Toc215653312)

**LIST OF TABLES**

[**Table S1.** Key fragment ions in MS/MS spectrum for rodriguesine A. 6](#_Toc215653313)

[**Table S2.** Neutral losses used to create the neutral loss graph (NLG). 9](#_Toc215653314)

[**Table S3.** Occurrence of minalemines’ *m/z* features in C_8_ SPE fractions. 10](#_Toc215653315)

[**Table S4.** NMR data for minalemines G (**1**) and H (**2**) in DMSO-*d*_6_. 13](#_Toc215653316)


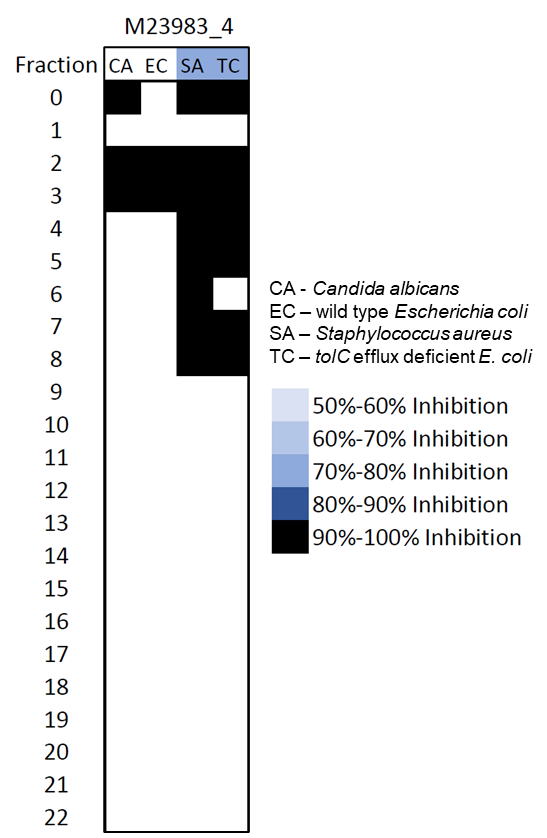


**Fig. S1**. Screening of subfractions generated for the antimicrobial campaign between NIAID and NCI.^1^

L. Martinez-Fructuoso, S. J. R. Arends, V. F. Freire, J. R. Evans, S. DeVries, B. D. Peyser, R. K. Akee, C. C. Thornburg, R. Kumar, S. Ensel, G. M. Morgan, G. D. McConachie, N. Veeder, L. R. Duncan, T. Grkovic and B. R. O'Keefe, *ACS Infect Dis*, 2023, **9**, 1245-1256.


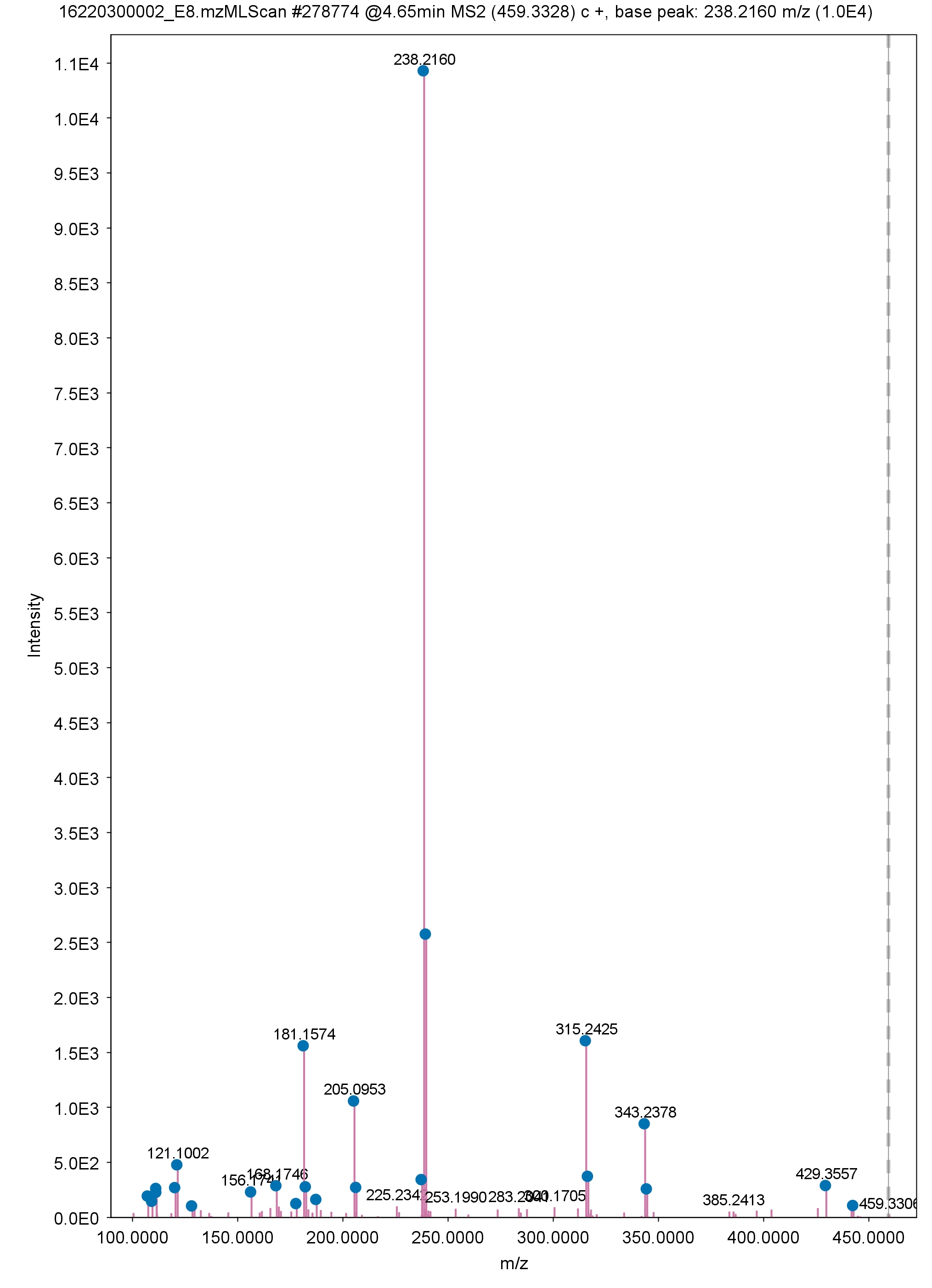


**Fig. S2.** MS^2^ spectrum extracted from the feature: *m/z* 459.3328, R_T_ 4.65 min.

**Table S1.** Key fragment ions in MS/MS spectrum for rodriguesine A.

| **Molecular Formula** | **Accurate mass** | **Relative intensity (%)** |
| --- | --- | --- |
| C_26_H_40_N_3_O_3_^+^ | 442.3065 | 1.03 |
| C_21_H_31_N_2_O_2_^+^ | 343.2381 | 8.15 |
| C_15_H_31_N_4_O_3_^+^ | 315.2425 | 15.40 |
| C_15_H_28_NO_3_^+^ | 238.2160 | 100.00 |
| C_11_H_13_N_2_O_2_^+^ | 205.0972 | 10.14 |

**
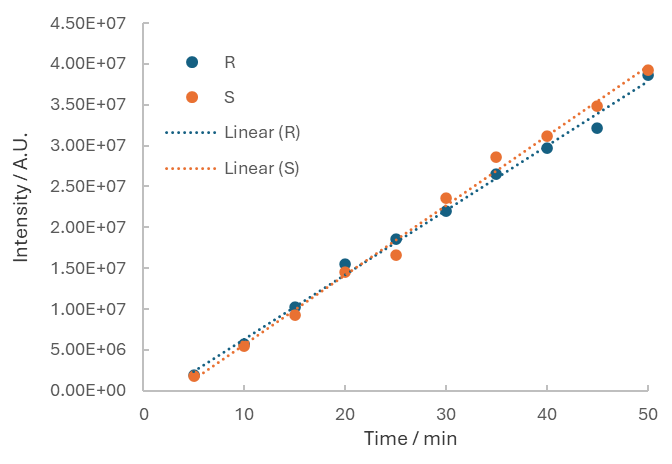
**

**Fig. S3.** Competing enantioselective conversion (CEC) reaction with *R*- and *S*-homobenzotetramisole (HBTM) coupled to LC-MS. Reaction rates of **1** treated with propionic anhydride using *R*- and *S*-HBTM catalysts.


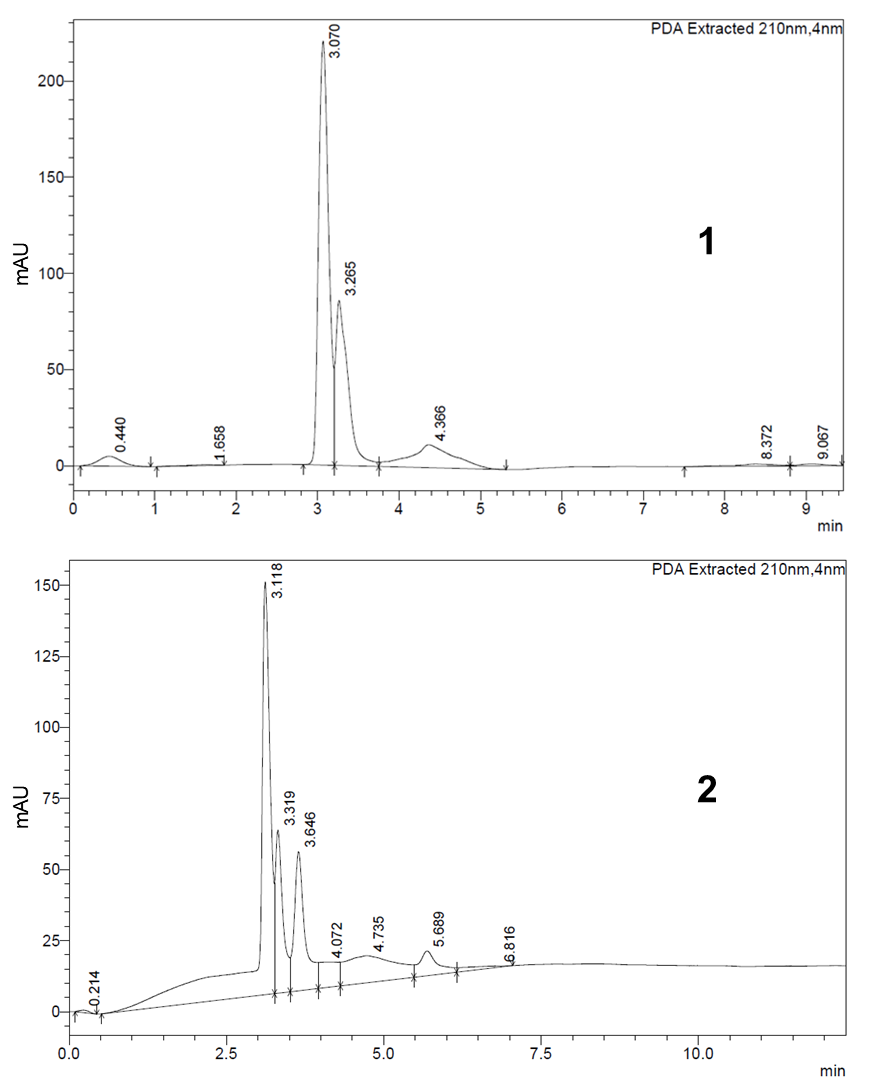


**Fig. S4.** Chromatogram of chiral analysis for minalemines G (**1**) and H (**2**).

**Table S2.** Neutral losses used to create the neutral loss graph (NLG).

| **Common Neutral Loss**  **(CNL)** | **Unique Neutral Loss for 1**  **(UNL1)** | **Unique Neutral Loss for 2**  **(UNL2)** |
| --- | --- | --- |
| 17.0265  NH_3_ | 186.1481  C_8_H_18_N_4_O | 179.0946  C_10_H_13_NO_2_ |
| 24.9952  CHN | 201.1589  C_8_H_19_N_5_O | 196.1212  C_10_H_16_N_2_O_2_ |
| 42.0217  CH_2_N_2_ | 203.1746  C_8_H_21_N_5_O | 221.1164  C_11_H_5_N_3_O_2_ |
| 130.1106  C_6_H_14_N_2_O | 228.1699  C_9_H_20_N_6_O | 351.2270  C_17_H_29_N_5_O_3_ |
| 155.1058  C_7_H_13_N_3_O |  |  |
| 172.1324  C_7_H_16_N_4_O |  |  |

**Table S3.** Occurrence of minalemines’ *m/z* features in C_8_ SPE fractions.

| ***m/z*** | **R_t_  (min)** | **Molecular Formula** | **Mass Error  (ppm)** | **Occurrence (fraction number)** |
| --- | --- | --- | --- | --- |
| 526.4550 | 3.41 | C_26_H_56_N_9_O_2_^+^ | -0.38 | 2 |
| 540.4703 | 3.68 | C_27_H_58_N_9_O_2_^+^ | -0.93 | 1 – 3 |
| 554.4860 | 3.82 | C_28_H_60_N_9_O_2_^+^ | -0.90 | 1 – 4, 6 |
| 568.5013 | 3.95 | C_29_H_62_N_9_O_2_^+^ | -1.41 | 1, 3, 6 |
| 606.4126 | 4.15 | C_26_H_56_N_9_O_5_S^+^ | 0.99 | 5 |
| 620.4276 | 4.61 | C_27_H_58_N_9_O_5_S^+^ | -0.16 | 1, 4, 5 |
| 634.4435 | 4.68 | C_28_H_60_N_9_O_5_S^+^ | 0.32 | 1, 4 – 6 |
| 477.3548 | 3.07 | C_25_H_45_N_6_O_3_^+^ | 0 | 2 |
| 505.3844 | 3.63 | C_27_H_49_N_6_O_3_^+^ | -3.36 | 3 – 5 |
| 519.4014 | 4.06 | C_28_H51N_6_O_3_^+^ | -0.58 | 3 – 5 |
| 531.3998 | 4.06 | C_29_H_51_N_6_O_3_^+^ | -3.58 | 4 |
| 533.4171 | 4.21 | C_29_H_53_N_6_O_3_^+^ | -0.56 | 1 – 6 |
| 547.4326 | 4.65 | C_30_H_55_N_6_O_3_^+^ | -0.73 | 1, 5, 6 |
| 561.4487 | 4.89 | C_31_H_57_N_6_O_3_^+^ | 0 | 5, 6 |


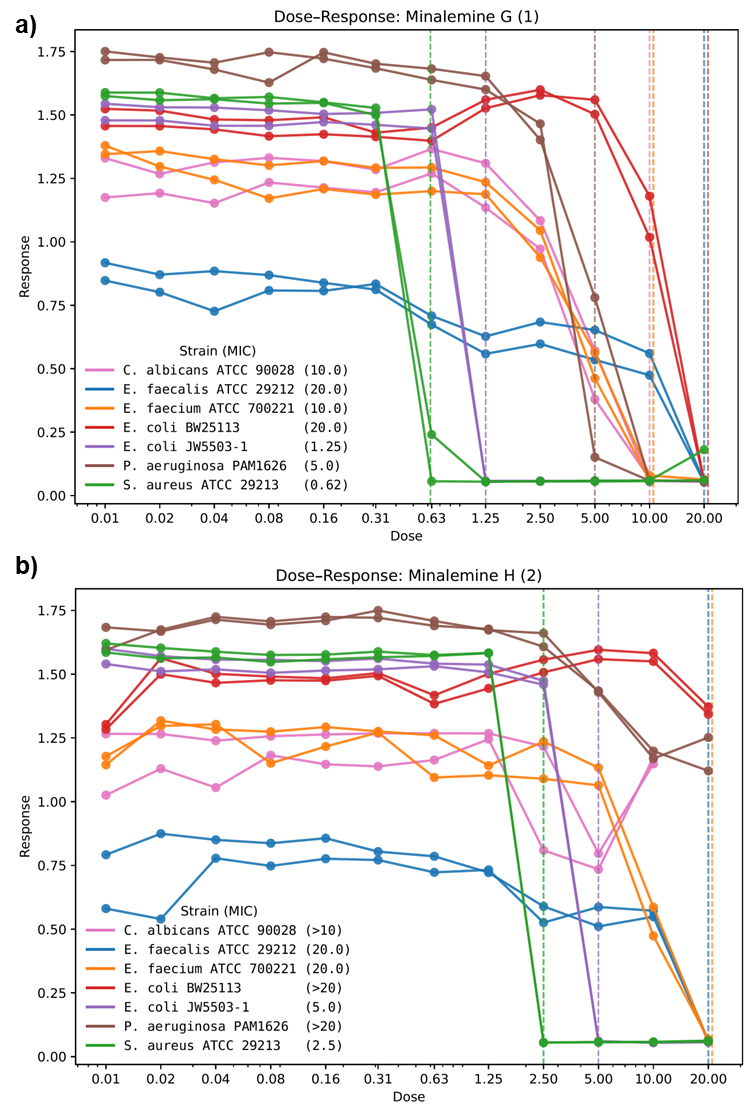


**Fig. S5.** Dose-response graphs of antimicrobial activity for minalemines G (a) and H (b).

**
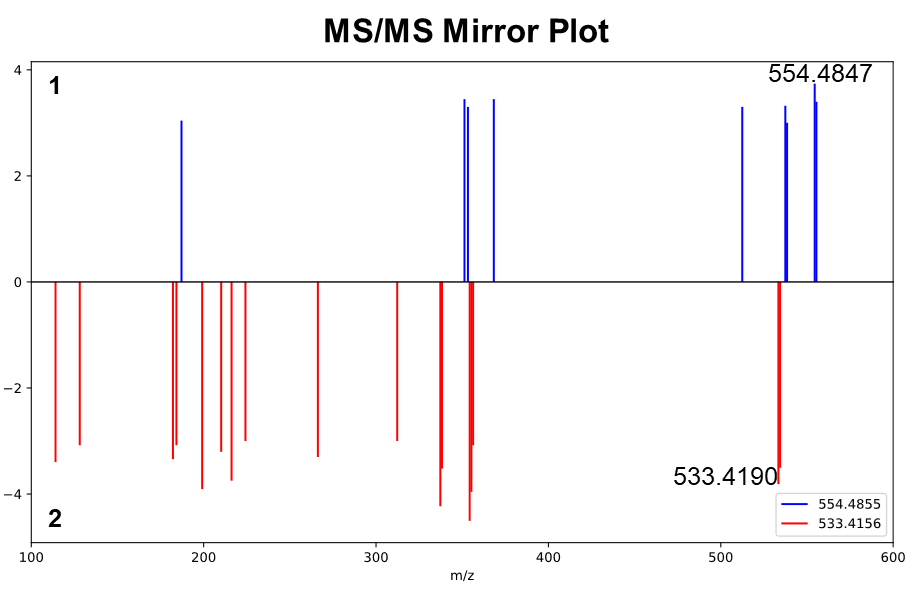
**

**Fig. S6.** Mirror plot of MS/MS data of minalemines G (**1**, top) and H (**2**, bottom).

**Fig. S7.** Synthetic compound reported by Maccari *et al*.^2^

G. Maccari, S. Sanfilippo, F. De Luca, D. Deodato, A. Casian, M. C. Dasso Lang, C. Zamperini, E. Dreassi, G. M. Rossolini, J. D. Docquier and M. Botta, *Bioorg Med Chem Lett*, 2014, **24**, 5525-5529.

**Table S4.** NMR data for minalemines G (**1**) and H (**2**) in DMSO-*d*_6_.

| **1**^a^ |  | | **2**^b^ | **1**^a^ | |
| --- | --- | --- | --- | --- | --- |
| Position | *δ*_C_^c^/*δ*_N_^d^, type | *δ*_H_^e^, mult (*J* in Hz) | Position | *δ*_C_^c^/*δ*_N_^d^, type | *δ*_H_^e^, mult (*J* in Hz) |
| *Hagma_1_* |  |  | *Hagma_1_* |  |  |
| 1 | 38.4, CH_2_ | 3.06, m | 1 | 38.4, CH_2_ | 3.06, m |
| 2 | 28.0, CH_2_ | 1.46, m | 2 | 28.0, CH_2_ | 1.46, m |
| 3 | 23.4, CH_2_ | 1.24, m | 3 | 23.4, CH_2_ | 1.24, m |
| 4 | 28.5, CH_2_ | 1.46, m | 4 | 28.5, CH_2_ | 1.46, m |
| 5 | 40.7, CH_2_ | 3.06, m | 5 | 40.7, CH_2_ | 3.06, m |
| 1-NH | 122.2, NH | 8.30, s | 1-NH | 122.2, NH | 8.30, s |
| 5-NH | 85.0, NH | 7.65, t (5.9) | 5-NH | 85.0, NH | 7.65, t (5.9) |
| C=N | 156.8, C | - | C=N | 156.8, C | - |
| *FA* |  |  | *FA* |  |  |
| 1 | 169.7, C | - | 1 | 169.7, C | - |
| 2 | 33.9, CH_2_ | 2.50^f^ | 2 | 33.9, CH_2_ | 2.50^f^ |
| 3 | 54.9, CH | 3.43, br s | 3 | 54.9, CH | 3.43, br s |
| 4 | 28.9, CH_2_ | 1.23, m | 4 | 28.9, CH_2_ | 1.23, m |
| 5-13 | 22.1 – 31.3, CH_2_ | 1.26, m | 5-13 | 22.1 – 31.3, CH_2_ | 1.26, m |
| 14 | 14.0, CH_3_ | 0.85, t (7.5) | 14 | 14.0, CH_3_ | 0.85, t (7.5) |
| *Gly* |  |  | *Gly* |  |  |
| 1 | 45.0, CH_2_ | 3.73, s | 1 | 45.0, CH_2_ | 3.73, s |
| 2 | 164.6, C | - | 2 | 164.6, C | - |
| NH-1 | - | 9.00, s  8.84, s | NH-1 | - | 9.00, s  8.84, s |
| *Hagma_2_* |  |  | *Hagma_2_* |  |  |
| 1 | 38.6, CH_2_ | 3.11, q (6.6) | 1 | 38.6, CH_2_ | 3.11, q (6.6) |
| 2 | 28.1, CH_2_ | 1.46, m | 2 | 28.1, CH_2_ | 1.46, m |
| 3 | 23.5, CH_2_ | 1.24, m | 3 | 23.5, CH_2_ | 1.24, m |
| 4  5 | 28.5, CH_2_  40.7, CH_2_ | 1.46, m  3.06, m | 4 | 28.5, CH_2_ | 1.46, m |
|  |  |  | 5 | 40.7, CH_2_ | 3.06, m |
| 1-NH | 117.3, NH | 8.43, s | 1-NH | 117.3, NH | 8.43, s |
| 5-NH | 85.0, NH | 7.65, t (5.9) | 5-NH | 85.0, NH | 7.65, t (5.9) |

^a^TFA salt; ^b^Free base; ^c^151 MHz; ^d^60 MHz; ^e^600 MHz; ^f^Superimposed by residual DMSO signal.

**
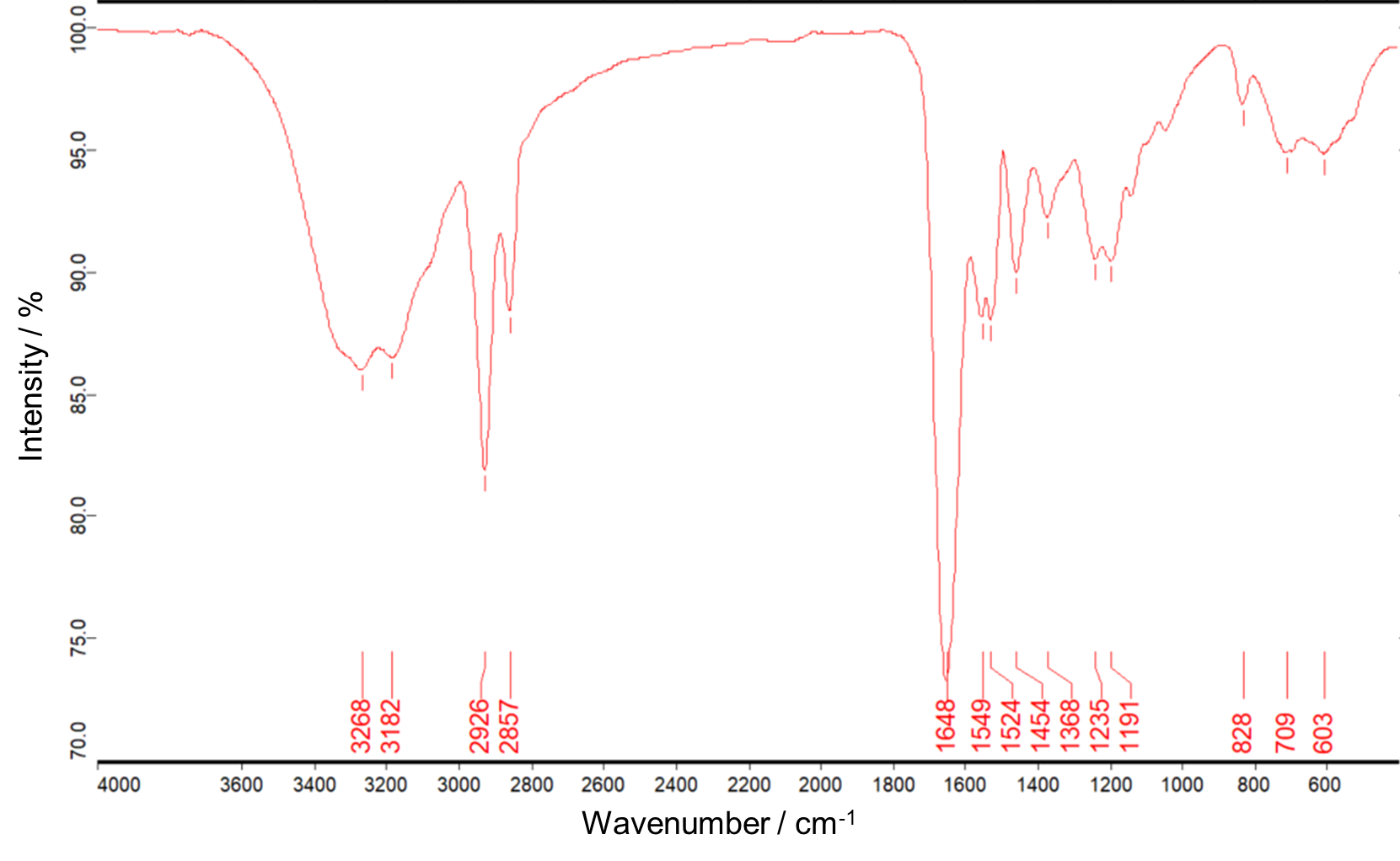
**

**Fig. S8.** FTIR spectrum of minalemide G (**1**).

**Fig. S9.** 600 MHz ^1^H NMR spectrum of minalemide G (**1**) in MeOH-*d*_4_. The spectrum was acquired over 4 scans using zg30 Bruker pulse sequence.

**Fig. S10**. Expansion of 600 MHz ^1^H NMR spectrum of minalemide G (**1**) in MeOH-*d*_4_. The spectrum was acquired over 4 scans using zg30 Bruker pulse sequence.

**Fig. S11**. Expansion of 600 MHz ^1^H NMR spectrum of minalemide G (**1**) in MeOH-*d*_4_. The spectrum was acquired over 4 scans using zg30 Bruker pulse sequence.

**Fig. S12**. Expansion of 600 MHz ^1^H NMR spectrum of minalemide G (**1**) in MeOH-*d*_4_. The spectrum was acquired over 4 scans using zg30 Bruker pulse sequence.

**Fig. S13.** Expansion of 600 MHz ^1^H NMR spectrum of minalemide G (**1**) in MeOH-*d*_4_. The spectrum was acquired over 4 scans using zg30 Bruker pulse sequence.

**Fig. S14**. 151 MHz ^13^C NMR spectrum of minalemide G (**1**) in MeOH-*d*_4_. The spectrum was acquired over 800 scans using zg0pg Bruker pulse sequence.

**Fig. S15**. ^1^H–^13^C HSQC NMR spectrum of minalemide G (**1**) in MeOH-*d*_4_. The spectrum was acquired over 2 scans using hsqcedetgpsisp2.3 Bruker pulse sequence.

**Fig. S16**. ^1^H–^1^H COSY NMR spectrum of minalemide G (**1**) in MeOH-*d*_4_. The spectrum was acquired over 2 scans using cosygpppqf Bruker pulse sequence.

**Fig. S17**. ^1^H–^13^C HMBC NMR spectrum of minalemide G (**1**) in MeOH-*d*_4_. The spectrum was acquired over 32 scans using hmbcgpl2ndqf Bruker pulse sequence.

**Fig. S18.** ^1^H–^15^N HMBC NMR spectrum of minalemide G (**1**) in MeOH-*d*_4_. The spectrum was acquired over 48 scans using hmbcgpndqf Bruker pulse sequence.

**Fig. S19.** 600 MHz ^1^H NMR spectrum of minalemide G (**1**) in DMSO-*d*_6_. The spectrum was acquired over 4 scans using zg30 Bruker pulse sequence.

**Fig. S20.** Expansion of 600 MHz ^1^H NMR spectrum of minalemide G (**1**) in DMSO-*d*_6_. The spectrum was acquired over 4 scans using zg30 Bruker pulse sequence.

**Fig. S21.** Expansion of 600 MHz ^1^H NMR spectrum of minalemide G (**1**) in DMSO-*d*_6_. The spectrum was acquired over 4 scans using zg30 Bruker pulse sequence.

**Fig. S22.** Expansion of 600 MHz 1H NMR spectrum of minalemide G (**1**) in DMSO-*d*_6_. The spectrum was acquired over 4 scans using zg30 Bruker pulse sequence.

**Fig. S23.** 151 MHz ^13^C NMR spectrum of minalemide G (**1**) in DMSO-*d*_6_. The spectrum was acquired over 6000 scans using zgpg30 Bruker pulse sequence.

**Fig. S24.** ^1^H–^13^C HSQC NMR spectrum of minalemide G (**1**) in DMSO-*d*_6_. The spectrum was acquired over 8 scans using hsqcedetgpsisp2.3 Bruker pulse sequence.

**Fig. S25.** ^1^H–^1^H COSY NMR spectrum of minalemide G (**1**) in DMSO-*d*_6_. The spectrum was acquired over 8 scans using cosygpppqf Bruker pulse sequence.

**Fig. S26.** ^1^H–^13^C HMBC NMR spectrum of minalemide G (**1**) in DMSO-*d*_6_. The spectrum was acquired over 96 scans using hmbcetgpl3nd Bruker pulse sequence.

**Fig. S27.** ^1^H–^15^N HSQC NMR spectrum of minalemide G (**1**) in DMSO-*d*_6_. The spectrum was acquired over 16 scans using hsqcetfpf3gpsi Bruker pulse sequence.

**Fig. S28.** ^1^H–^15^N HMBC NMR spectrum of minalemide G (**1**) in DMSO-*d*_6_. The spectrum was acquired over 128 scans using hmbcgpndqf Bruker pulse sequence.


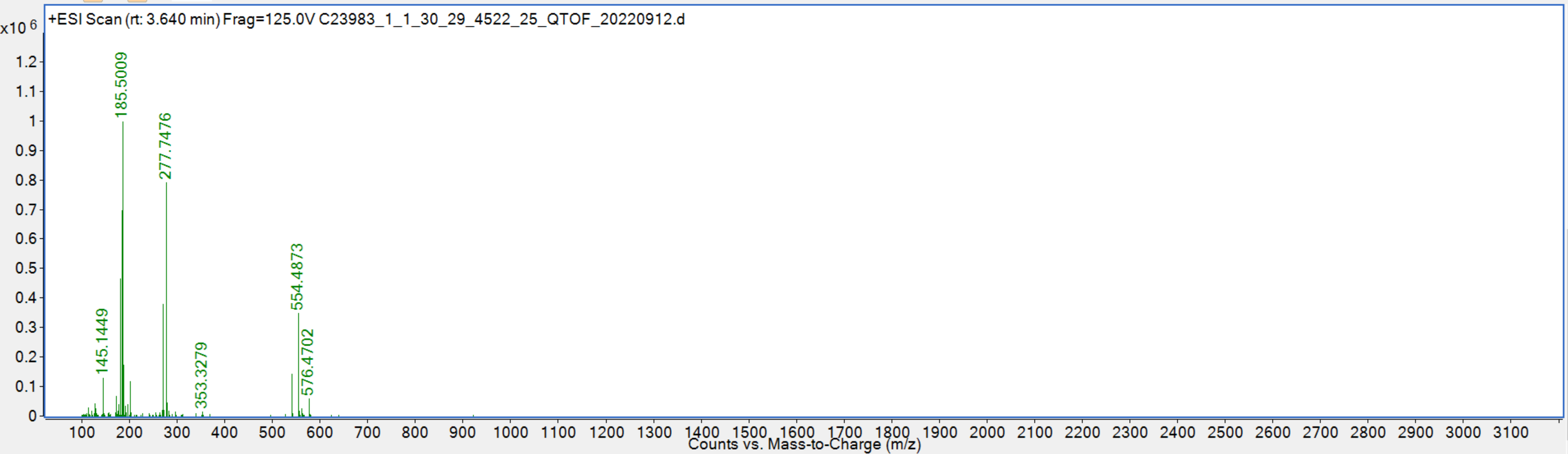


**Fig. S29**. HRESIMS spectrum of minalemide G (**1**).


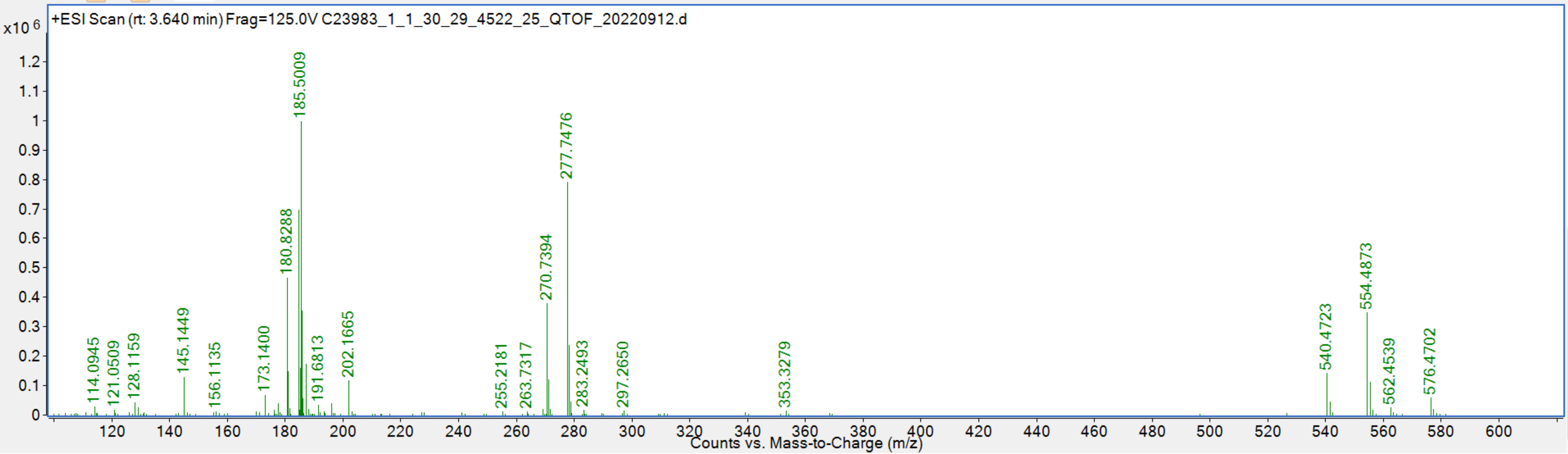


**Fig. S30**. Expansion of HRESIMS spectrum of minalemide G (**1**).


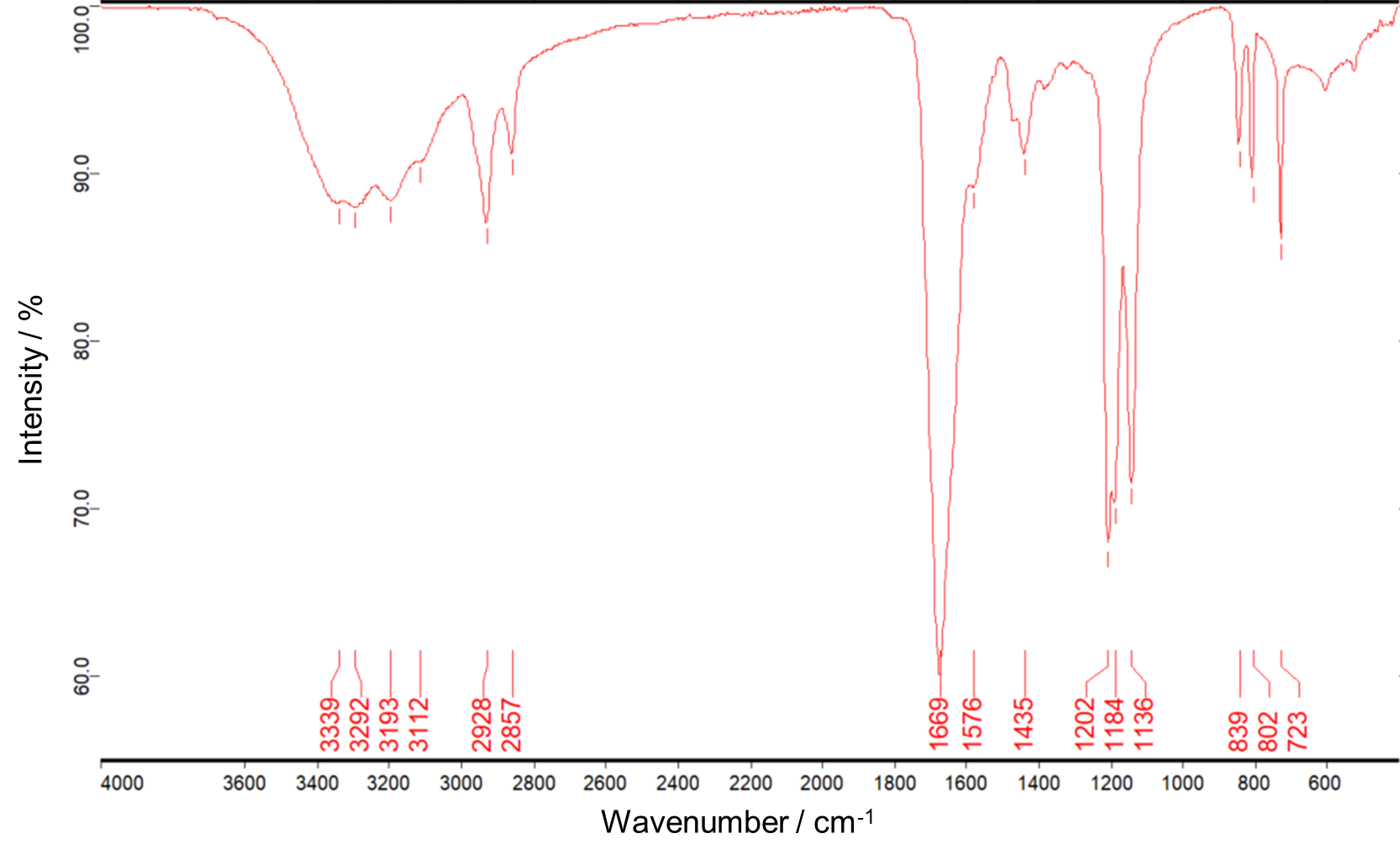


**Fig. S31.** FTIR spectrum of minalemine H (**2**).

**Fig. S32.** 600 MHz ^1^H NMR spectrum of minalemide H (**2**) in MeOH-*d*_4_. The spectrum was acquired over 128 scans using zg30 Bruker pulse sequence.

**Fig. S33.** Expansion of 600 MHz ^1^H NMR spectrum of minalemide H (**2**) in MeOH-*d*_4_. The spectrum was acquired over 128 scans using zg30 Bruker pulse sequence.

**Fig. S34.** Expansion of 600 MHz ^1^H NMR spectrum of minalemide H (**2**) in MeOH-*d*_4_. The spectrum was acquired over 128 scans using zg30 Bruker pulse sequence.

**Fig. S35.** Expansion of 600 MHz ^1^H NMR spectrum of minalemide H (**2**) in MeOH-*d*_4_. The spectrum was acquired over 128 scans using zg30 Bruker pulse sequence.

**Fig. S36.** 151 MHz ^13^C NMR spectrum of minalemide H (**2**) in MeOH-*d*_4_. The spectrum was acquired over 90000 scans using zg0pg Bruker pulse sequence.

**Fig. S37.** ^1^H–^13^C HSQC NMR spectrum of minalemide H (**2**) in MeOH-*d*_4_. The spectrum was acquired over 64 scans using hsqcedetgpsisp2.3 Bruker pulse sequence.

**Fig. S38.** ^1^H–^1^H COSY NMR spectrum of minalemide H (**2**) in MeOH-*d*_4_. The spectrum was acquired over 4 scans using cosygpppqf Bruker pulse sequence.

**Fig. S39.** ^1^H–^13^C HMBC NMR spectrum of minalemide H (**2**) in MeOH-*d*_4_. The spectrum was acquired over 96 scans using hmbcetgpl3nd Bruker pulse sequence.

**Fig. S40.** 600 MHz ^1^H NMR spectrum of minalemide H (**2**) in DMSO-*d*_6_. The spectrum was acquired over 4 scans using zg30 Bruker pulse sequence.

**Fig. S41.** Expansion of 600 MHz ^1^H NMR spectrum of minalemide H (**2**) in DMSO-*d*_6_. The spectrum was acquired over 4 scans using zg30 Bruker pulse sequence.

**Fig. S42.** Expansion of 600 MHz ^1^H NMR spectrum of minalemide H (**2**) in DMSO-*d*_6_. The spectrum was acquired over 4 scans using zg30 Bruker pulse sequence.

**Fig. S43.** Expansion of 600 MHz ^1^H NMR spectrum of minalemide H (**2**) in DMSO-*d*_6_. The spectrum was acquired over 4 scans using zg30 Bruker pulse sequence.

**Fig. S44.** 151 MHz ^13^C NMR spectrum of minalemide H (**2**) in DMSO-*d*_6_. The spectrum was acquired over 24000 scans using zgpg30 Bruker pulse sequence.

**Fig. S45.** ^1^H–^13^C coupled HSQC NMR spectrum of minalemide H (**2**) in DMSO-*d*_6_. The spectrum was acquired over 64 scans using hsqcedetgpsisp2.3_coupled Bruker pulse sequence.

**Fig. S46.** ^1^H–^1^H COSY NMR spectrum of minalemide H (**2**) in DMSO-*d*_6_. The spectrum was acquired over 6 scans using cosygpppqf Bruker pulse sequence.

**Fig. S47.** ^1^H–^13^C HMBC NMR spectrum of minalemide H (**2**) in DMSO-*d*_6_. The spectrum was acquired over 384 scans using hmbcetgpl3nd Bruker pulse sequence.

**
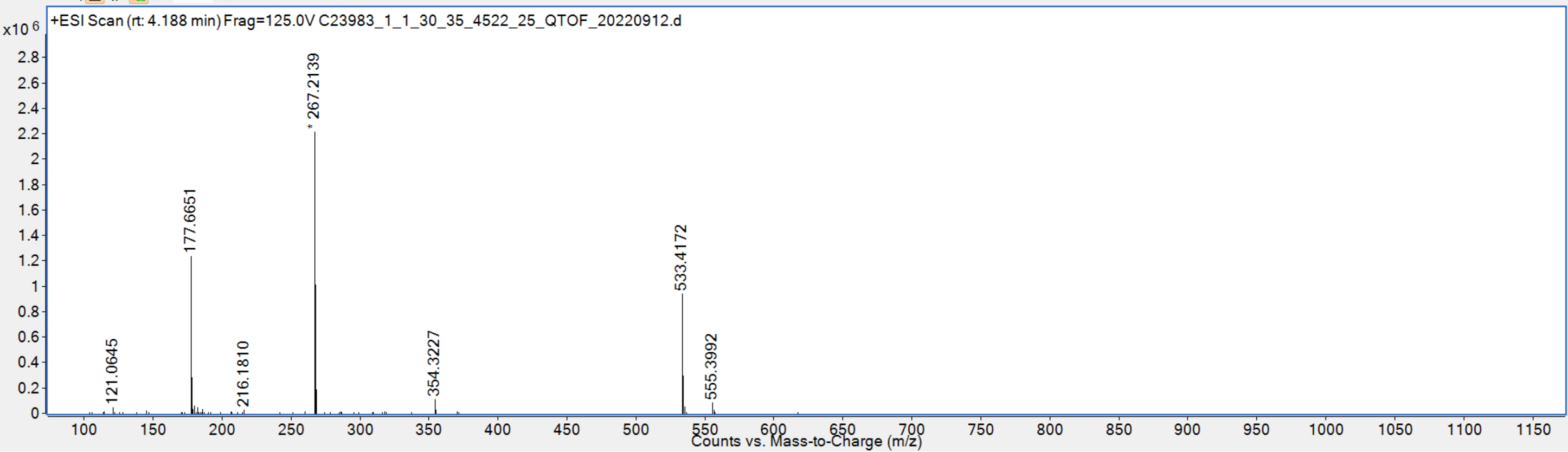
**

**Fig. S48.** HRESIMS spectrum of minalemide H (**2**).


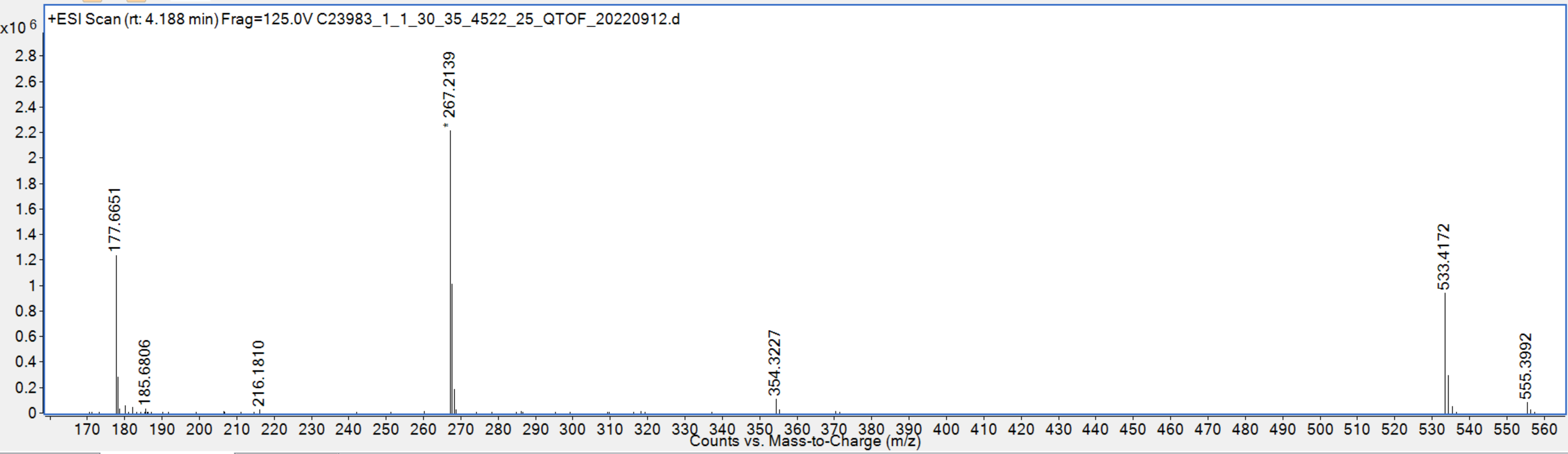


**Fig. S49.** Expansion of HRESIMS spectrum of minalemide H (**2**).


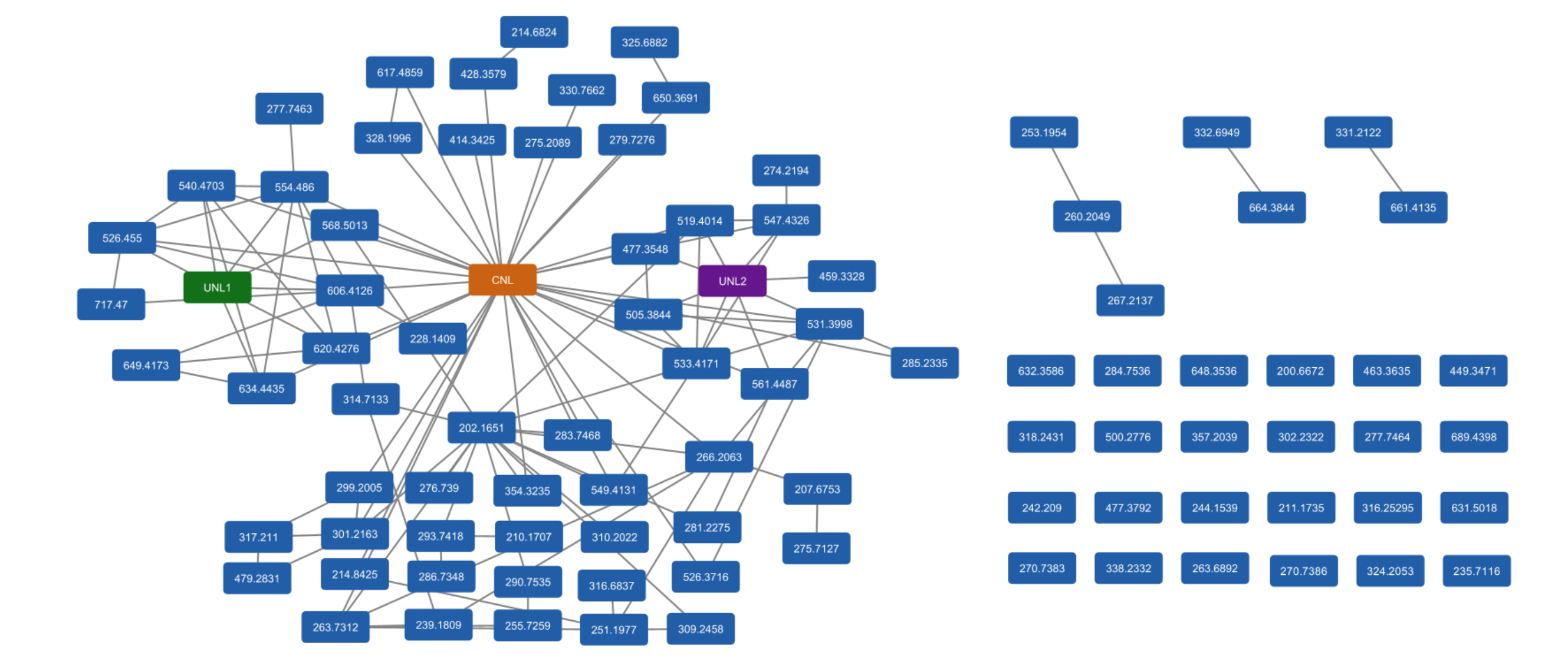


**Fig. S50.** Unfiltered neutral loss graph (NLG) of *Didemnum granulatum* fractions*.*
